# Evaluating Power-Law Scaling Factors for Harmonic Vibrational Frequencies across Model Chemistries

**DOI:** 10.1021/acs.jctc.6c01138

**Published:** 2026-08-15

**Authors:** Magnus W. D. Hanson-Heine, Adam M. Steer

**Affiliations:** School of Chemistry and Chemical Engineering, 7423 University of Southampton, Highfield, Southampton SO17 1BE, U.K.

## Abstract

Scaling factors are derived for fundamental harmonic vibrational frequencies and molecular thermodynamic corrections using the recently developed wavenumber power scaling (WPS) method [ Spectrochim. Acta, Part A 2026, 359, 127967 10.1016/j.saa.2026.12796742035662]. WPS enables a simple two-parameter scaling of vibrational frequencies that improves on uniform scaling errors by up to ca. 64% for the best methods tested. Power law and uniform scaling factors are calculated using over 500 model chemistries, including wave function theories (HF, MP2, CCSD) and density functional theories with a range of atom-centered basis sets. Experimental comparisons are performed using a set of 894 vibrations with C≡C and C≡N triple bond bending modes removed. The B97, B97–1, and τ-HCTHh density functional methods match the experimental fundamentals closely and are recommended for general use. WPS frequencies converge more quickly with respect to basis-set size than uniformly scaled frequencies, making them both more accurate and computationally efficient to calculate. Power law scaling describes fundamental vibrations, enthalpies, and entropies using a unified set of scaling factors, and can improve generalized gradient approximation frequencies that are resistant to uniform scaling.

## Graphical abstract

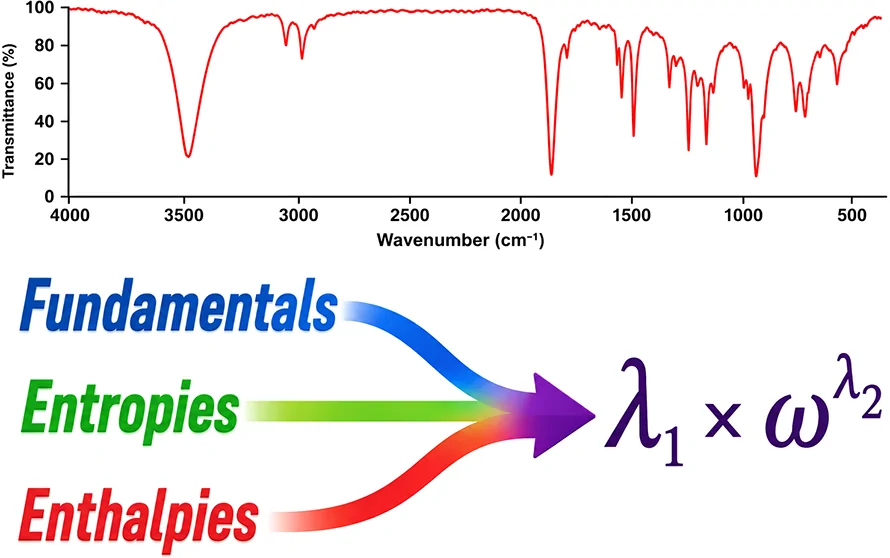

## Introduction

Quantum chemical harmonic vibrational frequencies^1^ deviate from experimentally observed vibrational transitions for many reasons, including anharmonic effects, basis-set deficiencies, and incomplete descriptions of electron correlation. Pople and co-workers proposed an empirical correction to account for these deviations by multiplying the harmonic energies using a single uniform scaling factor.^2^ Multiplicative scaling factors have since been derived for many electronic structure theories,^3–43^ variously called linear scaling, constant scaling, or uniform scaling factors. Within this approach, the fundamental molecular transition frequencies from the ground state to the first excited state dominated by each normal mode become

1 $$\nu_{i}={\lambda \omega }_{i}$$

where ν_*i*_ is the *i*th experimentally observed fundamental frequency, ω_*i*_ is the calculated harmonic frequency, and λ is the scaling factor. However, as harmonic deviations are not constant across the spectrum, alternative corrections have been proposed, including separate scaling factors for different spectral regions,^18^ inverse wavenumber scaling at lower frequencies,^30,31^ and polynomial scaling functions.^44–51^ Many of these approaches introduce discontinuities,^16,18^ inflections at polynomial vertices,^51^ and risk under or overfitting leading to inconsistent results across model chemistries.^10,51^ Recently, the wavenumber power scaling (WPS) method was developed,^51^ showing that the underlying scaling factor function is generally monotonically decreasing and can be well represented using a power law curve. This gives a simple two-parameter formula for the fundamental frequencies

2 $$\nu_{i}=\lambda_{1}\omega_{i}^{\lambda_{2}}$$

which treats the anharmonicity consistently across the entire spectral range while being more accurate and stable across electronic structure methods.^51^ More complex scaling schemes also exist. Pulay and co-workers developed scaled quantum mechanical (SQM) force-fields,^52–55^ Borowski developed effective scaling frequency factors (ESFFs),^56–60^ Irikura developed mass scaling,^61^ partial anharmonic frequencies can be scaled,^62^ and machine learning methods can predict molecular anharmonicity.^63–68^ However, these approaches use additional chemical information, often require specialized software, and cannot be readily applied to existing harmonic frequencies. In contrast, power law scaling can be applied to any existing or published harmonic frequency using just a basic calculator. Here we derive and evaluate power law scaling factors for fundamental vibrational transition frequencies, thermal enthalpy contributions, and thermal entropy contributions, across a wide range of molecules and electronic structure theories, providing benchmark data and recommendations for their wider use.

Frequency-region-specific approaches can also be used with WPS by introducing additional parameters and discontinuities into the power law scaling function. However, previous calculations have found that global WPS outperformed regional uniform scaling across all of the regions tested, while the best-performing 8-parameter regional WPS method only improved on global WPS by ca. 0.5 cm^–1^ on average.^51^ This led the author to note that the benefits of region-specific scaling may be an artifact of needing additional fitting parameters to compensate for using scaling functions that do not closely match the underlying scaling function’s shape, and region-specific scaling has not been investigated further here.

## Computational Details

Optimized molecular geometries and harmonic vibrational frequencies were calculated using a developmental version of the Q-Chem 6 quantum chemical software package.^69^ Electronic structures were calculated using Hartree–Fock theory (HF),^70–72^ second-order Møller–Plesset perturbation theory (MP2),^73^ and coupled cluster theory with single and double excitations (CCSD).^74^ Both of the correlated wave function methods employed frozen core orbitals. Electronic structures were also calculated using density functional theory (DFT) with exchange-correlation functionals parametrized using the generalized gradient approximation (GGA), including BLYP,^75,76^ BP86,^75,77^ EDF1,^78^ HCTH-93,^79^ HCTH-120,^80^ HCTH-147,^80^ HCTH-407,^81^ KT3,^82^ OLYP,^83^ and PBE,^84^ using the meta-GGA, including B97M-V,^85^ rSCAN,^86^ SCAN,^87^ τ-HCTH,^88^ TPSS,^89^ and VSXC,^90^ using the global hybrid-GGA, including B1LYP,^91^ B3LYP,^92,93^ B3P86,^77,94^ B3PW91,^92^ B97,^95^ B97–K,^96^ B97–1,^79^ B97–2,^97^ B97–3,^98^ BHHLYP,^76,99^ EDF2,^100^ MPW1K,^101^ MPW1PW91,^102^ O3LYP,^103^ PBE0,^104^ and X3LYP,^105^ using the meta-hybrid-GGA, including M05,^106^ M05–2X,^107^ M06,^108^ M06–2X,^108^ M08-HX,^109^ M08-SO,^109^ τ-HCTHh,^88^ and TPSS0,^110^ and using the range-separated hybrid-GGA, including CAM-B3LYP,^111^ HSE-HJS,^112,113^ LC-rVV10,^114^ ωB97X-D,^115^ ωB97X-D3,^116^ and ωB97X-V.^117^ We have initially combined these methods with the Pople^118–122^ and Dunning^123,124^ type atom-centered electronic basis sets 6–31G­(d), 6–31+G­(d,p), 6–31++G­(d,p), 6–31+G­(2df,p), 6–311+G­(d,p), 6–311++G­(d,p), 6–311+G­(2df,p), 6–311++G­(2df,p), cc-pVTZ, and aug-cc-pVTZ, and then combined a subset of the best-performing methods with an extended range of basis sets, including 6–311++G­(3df,3pd), pc-2,^125^ pc-3,^125^ aug-pc-2,^126^ aug-pc-3,^126^ Def2-TZVP,^127^ Def2-TZVPD,^128^ cc-pVDZ, cc-pVQZ, aug-cc-pVDZ, and aug-cc-pVQZ.

Recent work by Hanson-Heine has demonstrated that C≡C and C≡N triple bond bending vibrational modes can be pathological for scaling factors,^51^ so uniform scaling factors and power law scaling factors have been derived using the experimental frequencies in Hanson-Heine’s 894 mode modification of the F1 frequency set,^51^ which has multireference,^13,14^ internal rotation, inversion, and triple bond bending modes removed.^51^ More modern vibrational frequency sets have been developed,^129,130^ but have not yet been benchmarked with respect to the removal of triple bond bending modes. The modified F1 set was chosen to aid comparisons with the existing literature, and has been used with corrected assignments adopted from the expanded VIBFREQ1295 frequency set proposed by Zapata and McKemmish.^129^ The details of these experimental frequencies are available in the Supporting Information. The data set contains 321 low frequency modes below 1000 cm^–1^, 467 mid frequency modes between 1000 and 3000 cm^–1^, and 106 high frequency modes above 3000 cm^–1^. However, data becomes sparser at lower frequencies, which can cause predictions to be less transferable when considering very low frequency modes. Merrick et al. found that the NO_2_, ClCCCl, HCCCl, HCCCCH, SiH_3_CCH, CH_3_COCH_3_, and CH_3_CCCH_3_ molecules needed to be removed from MP2 calculations in order to produce meaningful scaling factors,^31^ and this approach has been followed here. Similarly, CCSD calculations of the ClCCCl and HCCCCH molecules showed convergence issues for some methods and have been removed from the CCSD calculations of scaling factors. To test the transferability of some of the best-performing scaling factors, scaled frequencies have been calculated for the benzonitrile and naphthalene molecules and cross-validation has been performed by randomly splitting the modified F1 set of molecules into 100 training and test sets with set size ratios of 9:1, 8:2, and 7:3, calculating power law scaling factors and scaled frequency errors for each of these training data partitions, and then applying the scaling factors to each of the test sets. The default Q-Chem integration grids have been used for each method. However, larger grids were necessary to converge some of the CH_3_COOH and HCOOCH_3_ molecules, particularly for the meta-GGAs, and in these cases, the Euler-Maclaurin-Lebedev EML-(99,590) integration grid was used. General discussions of integration grids can be found elsewhere,^131–133^ and the details of the specific grids used in this study are available in the Supporting Information. Mean absolute deviations (MADs), median absolute deviations (MEDs), and root mean squared deviations (RMSDs) are reported here for the differences between the scaled and experimental values, and the scaling factor coefficients are reported to 6 decimal places in the main text, with higher accuracy available in the Supporting Information. Vibrational modes have been numbered in order of their ascending energy for each molecule.

Uniform fundamental scaling factors have been calculated by minimizing the residuals with respect to the set of *M* experimental frequencies^30^

3 $$\xi =\sum_{i=1}^{M}{\left(\lambda \omega_{i}-\nu_{i}\right)}^{2}$$

giving an expression for the scaling factors as

4 $$\lambda =\frac{\sum_{i=1}^{M}\left(\omega_{i}\nu_{i}\right)}{\sum_{i=1}^{M}{\left(\omega_{i}\right)}^{2}}$$

Power law fundamental frequency scaling factors have been determined by minimizing the equivalent log–log residual using the *“LINEST”* function in the Microsoft Excel software^134^ and using Python libraries for the cross-validation

5 $$\xi =\sum_{i=1}^{M}{\left(\ln\left(\lambda_{1}\omega_{i}^{\lambda_{2}}\right)-\ln\left(\nu_{i}\right)\right)}^{2}$$

The fundamental vibrational frequencies in wavenumbers can also be used to calculate thermal vibrational contributions to molecular enthalpies and entropies by approximating them across the frequency set as^31^

6 $$\Delta H_{\mathrm{vib}}\left(\nu ,T\right)=N_{A}\mathrm{hc}\left(\frac{\nu_{i}}{\exp\left(\frac{\mathrm{hc}\nu_{i}}{k_{B}T}\right)-1}\right)$$

and

7 $$S_{vib}\left(\nu ,T\right)=R\left(\frac{\frac{\mathrm{hc}\nu_{i}}{k_{B}T}}{\exp\left(\frac{\mathrm{hc}\nu_{i}}{k_{B}T}\right)-1}-ln\left(1-e\mathrm{xp}\left(-\frac{\mathrm{hc}\nu_{i}}{k_{B}T}\right)\right)\right)$$

where *N*_A_, *h*, *k*_B_, *c*, and *T* are Avogadro’s constant, Planck’s constant, Boltzmann’s constant, the speed of light in cm/s, and the temperature, respectively.

Specific uniform and WPS scaling factors for these vibrational enthalpy and entropy contributions have been determined by minimizing the residuals

8 $$\xi =\sum_{i=1}^{M}{\left(\Delta H_{v\mathrm{ib}}^{\mathrm{calc}}\left(\nu_{i},T\right)-\Delta H_{\mathrm{vib}}^{\exp}\left(\nu_{i},T\right)\right)}^{2}$$

and

9 $$\xi =\sum_{i=1}^{M}{\left(S_{vib}^{calc}\left(T\right)-S_{vib}^{\exp}\left(T\right)\right)}^{2}$$

where the calculated enthalpy and entropy values are obtained by substituting either the uniformly scaled transitions from eq 1, or the WPS transitions from eq 2, into both eqs 6 and 7, respectively.

## Results and Discussion

### Fundamental Frequencies

Fundamental harmonic frequency scaling factors and the scaled frequency deviations from experiment are presented in Tables 1, 2, 3, and 4 and the Supporting Information, with select trends shown in Figure 1. The B97 and B97–1 functionals perform best out of the hybrid DFT methods, giving WPS RMSDs of 16.1 and 16.2 cm^–1^ with the 6–311+G­(2df,p) basis set, respectively, compared to uniform scaling RMSDs of 25.3 and 25.0 cm^–1^. This is particularly noteworthy as meta-analyses have found that uniform scaling rarely performs better than a 25 cm^–1^ RMSD irrespective of the model chemistry chosen.^19^ The accuracy ordering of these functionals also swaps depending on which scaling method is used, with B97–1 found to be superior for uniform scaling and B97 found to be superior for WPS. The meta-hybrid τ-HCTHh functional then further reduces the WPS RMSD to 15.8 cm^–1^ when combined with the 6–311++G­(2df,p) basis set.

**Figure 1:**
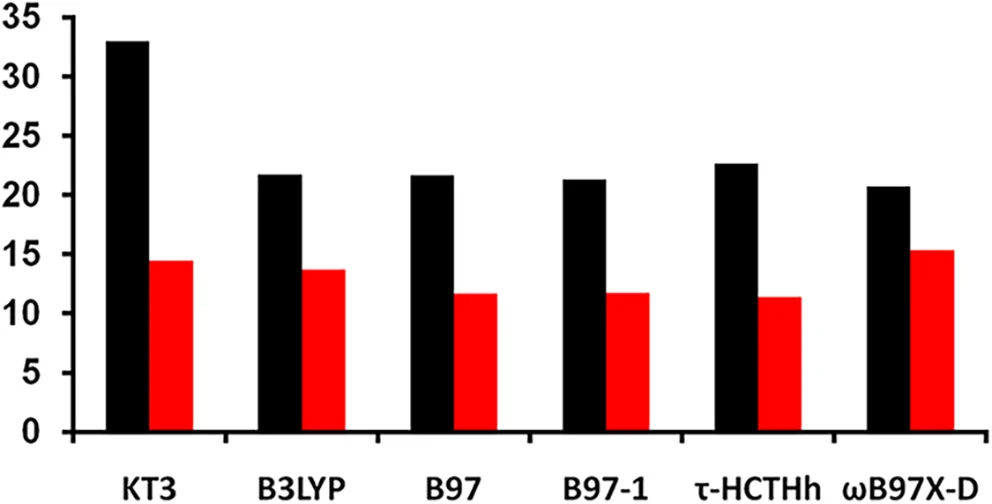
Uniform scaling (black) and power law scaling (red) MADs (in cm^–1^) for select functionals with the aug-cc-pVTZ basis.

**Table 1:** Power-Law Scaling Factors for Fundamental Vibrations

method	6–31G(d)		6–31+G(d,p)		6–31+G(2df,p)		6–311+G(d,p)		6–311+G(2df,p)	
	λ_1_	λ_2_	λ_1_	λ_2_	λ_1_	λ_2_	λ_1_	λ_2_	λ_1_	λ_2_
HF	0.968040	0.989248	0.975164	0.989350	0.944087	0.994225	0.954134	0.992873	0.919194	0.997825
MP2^a^	1.166903	0.972569	1.265319	0.962298	1.192636	0.971144	1.198879	0.970679	1.142416	0.977308
CCSD^b^	1.120337	0.978399	1.211427	0.967696	1.130783	0.977488	1.137835	0.977224	1.075350	0.984647
BLYP	1.371789	0.957753	1.440015	0.952336	1.376515	0.958639	1.441871	0.952506	1.358070	0.960246
BP86	1.313665	0.963211	1.367561	0.958830	1.317463	0.964173	1.360458	0.959992	1.155322	0.977327
EDF1	1.303863	0.962760	1.359795	0.958131	1.312097	0.963265	1.351972	0.959339	1.286200	0.965870
HCTH-93	1.271341	0.965486	1.322399	0.961200	1.278826	0.966081	1.310211	0.962883	1.247688	0.969360
HCTH-120	1.282412	0.964505	1.335439	0.960117	1.287086	0.965431	1.325218	0.961541	1.257735	0.968444
HCTH-147	1.281578	0.964476	1.333555	0.960161	1.286304	0.965379	1.322174	0.961720	1.256217	0.968483
HCTH-407	1.261484	0.965904	1.313435	0.961569	1.269285	0.966598	1.303246	0.963017	1.240234	0.969666
KT3	1.303994	0.960445	1.351493	0.956592	1.308266	0.961247	1.345545	0.957633	1.292200	0.962996
OLYP	1.286631	0.964328	1.343155	0.959638	1.301491	0.964288	1.333840	0.961015	1.271792	0.967367
PBE	1.298955	0.964303	1.353107	0.959904	1.307130	0.964914	1.344907	0.961183	1.282928	0.967376
B97M-V	1.164994	0.974076	1.213900	0.969321	1.158173	0.975854	1.189704	0.972403	1.124476	0.979842
rSCAN	1.199800	0.971229	1.243115	0.967420	1.195232	0.973021	1.222698	0.970087	1.161361	0.976821
SCAN	1.215125	0.969246	1.251218	0.966241	1.201760	0.971904	1.228903	0.969086	1.166006	0.975844
τ-HCTH	1.276294	0.964936	1.326324	0.960683	1.275390	0.966279	1.304675	0.963411	1.242383	0.969848
TPSS	1.336786	0.958491	1.384860	0.954680	1.332415	0.960292	1.363669	0.957341	1.302964	0.963308
VSXC	1.348906	0.956778	1.402310	0.952574	1.337198	0.959258	1.381263	0.955082	1.294066	0.963751
B1LYP	1.198722	0.970420	1.237476	0.967243	1.191015	0.972707	1.222976	0.969283	1.166295	0.975406
B3LYP	1.217240	0.969138	1.258469	0.965749	1.211171	0.971212	1.245463	0.967594	1.187153	0.973779
B3P86	1.184657	0.972593	1.220232	0.969647	1.181098	0.974412	1.202897	0.972082	1.155322	0.977327
B3PW91	1.184888	0.972238	1.220616	0.969252	1.183391	0.973816	1.202969	0.971736	1.156636	0.976863
B97	1.206444	0.970536	1.244420	0.967410	1.204829	0.972121	1.228419	0.969642	1.178036	0.975098
B97–K	1.147715	0.974365	1.174956	0.972315	1.138877	0.976902	1.159565	0.974624	1.115251	0.979713
B97–1	1.201484	0.970893	1.238242	0.967891	1.198939	0.972585	1.222080	0.970145	1.172511	0.975524
B97–2	1.176472	0.972404	1.211232	0.969483	1.173793	0.974123	1.190686	0.972306	1.143587	0.977637
B97–3	1.162347	0.973654	1.193038	0.971177	1.157450	0.975644	1.177892	0.973406	1.130697	0.978746
BHHLYP	1.087862	0.978649	1.110115	0.977011	1.071681	0.982135	1.088507	0.980170	1.045764	0.985362
EDF2	1.200702	0.970719	1.240943	0.967379	1.195738	0.972689	1.225192	0.969550	1.169919	0.975507
MPW1K	1.087879	0.979311	1.111296	0.977466	1.080054	0.981774	1.088109	0.980870	1.053954	0.985090
MPW1PW91	1.159507	0.974098	1.193464	0.971255	1.158583	0.975669	1.173177	0.974106	1.130634	0.978955
O3LYP	1.215683	0.969607	1.258945	0.965991	1.222443	0.970373	1.244148	0.968048	1.193661	0.973555
PBE0	1.153192	0.975023	1.185298	0.972392	1.153073	0.976544	1.168089	0.974895	1.128478	0.979444
X3LYP	1.205469	0.970126	1.245678	0.966828	1.198901	0.972293	1.231536	0.968816	1.174237	0.974967
M05	1.154085	0.974901	1.185260	0.972543	1.158185	0.975933	1.157346	0.976399	1.119341	0.980812
M05–2X	1.154953	0.973037	1.172887	0.972002	1.135290	0.976721	1.151136	0.974829	1.108993	0.979684
M06	1.147008	0.976857	1.183676	0.973688	1.141042	0.978796	1.156887	0.977253	1.103770	0.983206
M06–2X	1.136279	0.975973	1.156897	0.974593	1.118093	0.979519	1.137009	0.977209	1.096398	0.982024
M08-HX	1.135982	0.977058	1.162915	0.974924	1.129454	0.979057	1.146470	0.977188	1.105099	0.982032
M08-SO	1.150149	0.975711	1.173375	0.974265	1.140273	0.978367	1.146202	0.978118	1.110850	0.982156
τ-HCTHh	1.216724	0.969966	1.256712	0.966611	1.213310	0.971666	1.237626	0.969143	1.186178	0.974641
TPSS0	1.174994	0.971185	1.204181	0.968850	1.166393	0.973604	1.178918	0.972299	1.137049	0.977065
CAM-B3LYP	1.133159	0.976735	1.166326	0.974002	1.126372	0.979063	1.148520	0.976545	1.102175	0.981874
HSE-HJS	1.156680	0.974703	1.188887	0.972074	1.155954	0.976289	1.172256	0.974500	1.131099	0.979208
LC-rVV10	1.065847	0.982250	1.090396	0.980284	1.067728	0.983684	1.070250	0.983353	1.043433	0.986786
ωB97X-D	1.125542	0.977783	1.158449	0.974924	1.121261	0.979752	1.140641	0.977568	1.096283	0.982783
ωB97X-D3	1.117934	0.978323	1.148806	0.975704	1.113807	0.980325	1.130107	0.978476	1.088722	0.983411
ωB97X-V	1.115447	0.978586	1.145197	0.976130	1.113682	0.980369	1.126661	0.978857	1.087662	0.983539

a MP2 problem cases were removed.

b ClCCCl and HCCCCH were removed.

**Table 2:** Power-Law Scaling Factors for Fundamental Vibrations (cont.)

method	6–31++G(d,p)		6–311++G(d,p)		6–311++G(2df,p)		cc-pVTZ		aug-cc-pVTZ	
	λ_1_	λ_2_	λ_1_	λ_2_	λ_1_	λ_2_	λ_1_	λ_2_	λ_1_	λ_2_
HF	0.975482	0.989322	0.954477	0.992832	0.919048	0.997854	0.929799	0.996273	0.935918	0.995523
MP2^a^	1.277714	0.961152	1.214851	0.969060	1.143766	0.977180	1.158412	0.975268	1.189365	0.972262
CCSD^b^	1.222118	0.966662	1.182263	0.972069	1.076200	0.984566	1.095533	0.981980		
BLYP	1.439459	0.952413	1.443375	0.952387	1.357929	0.960274	1.363207	0.959616	1.386022	0.957602
BP86	1.366976	0.958912	1.362015	0.959859	1.293633	0.966563	1.302700	0.965541	1.319665	0.963998
EDF1	1.358888	0.958250	1.353106	0.959249	1.285888	0.965919	1.292083	0.965213	1.311026	0.963468
HCTH-93	1.321760	0.961297	1.311748	0.962748	1.247422	0.969403	1.253497	0.968717	1.270536	0.967099
HCTH-120	1.334777	0.960210	1.326116	0.961467	1.257656	0.968464	1.262309	0.967908	1.280819	0.966154
HCTH-147	1.332861	0.960258	1.325052	0.961447	1.256209	0.968496	1.261855	0.967845	1.279200	0.966205
HCTH-407	1.312722	0.961666	1.304286	0.962927	1.240270	0.969670	1.242175	0.969442	1.260115	0.967731
KT3	1.350562	0.956716	1.346893	0.957519	1.292235	0.963001	1.293343	0.962554	1.313208	0.960798
OLYP	1.341825	0.959807	1.334723	0.960951	1.271584	0.967405	1.271156	0.967441	1.291866	0.965532
PBE	1.352835	0.959962	1.346150	0.961079	1.283208	0.967361	1.287135	0.966906	1.305503	0.965227
B97M-V	1.213646	0.969373	1.190703	0.972305	1.124397	0.979862	1.140257	0.977702	1.153772	0.976260
rSCAN	1.242855	0.967471	1.223872	0.969978	1.161370	0.976832	1.173927	0.975283	1.186483	0.974020
SCAN	1.251592	0.966215	1.229912	0.968991	1.166677	0.975776	1.172418	0.975182	1.185826	0.973751
τ-HCTH	1.325458	0.960798	1.305795	0.963316	1.242392	0.969860	1.258369	0.968001	1.272041	0.966697
TPSS	1.384191	0.954773	1.364837	0.957248	1.302915	0.963329	1.312385	0.962319	1.328717	0.960822
VSXC	1.400646	0.952765	1.382804	0.954953	1.293965	0.963773	1.299672	0.962935	1.312151	0.961841
B1LYP	1.237075	0.967307	1.223887	0.969197	1.166334	0.975412	1.176808	0.974108	1.190998	0.972681
B3LYP	1.258020	0.965817	1.246466	0.967500	1.187106	0.973795	1.197227	0.972559	1.211845	0.971112
B3P86	1.220080	0.969684	1.204031	0.971970	1.155317	0.977341	1.166552	0.975984	1.178453	0.974792
B3PW91	1.220336	0.969306	1.203852	0.971653	1.155317	0.977341	1.167392	0.975597	1.179355	0.974402
B97	1.244086	0.967470	1.229379	0.969553	1.178034	0.975111	1.186771	0.974107	1.200326	0.972766
B97–K	1.174856	0.972348	1.160347	0.974546	1.115232	0.979725	1.123604	0.978716	1.135609	0.977467
B97–1	1.237797	0.967963	1.223094	0.970048	1.172534	0.975533	1.181607	0.974486	1.194689	0.973189
B97–2	1.210732	0.969563	1.191511	0.972229	1.143535	0.977655	1.155063	0.976255	1.166956	0.975044
B97–3	1.193061	0.971195	1.178913	0.973303	1.130705	0.978756	1.143087	0.977251	1.154991	0.976026
BHHLYP	1.109972	0.977047	1.089038	0.980114	1.045532	0.985402	1.057223	0.983834	1.066829	0.982775
EDF2	1.240585	0.967439	1.226077	0.969468	1.169336	0.975583	1.180892	0.974180	1.194573	0.972815
MPW1K	1.111079	0.977513	1.088692	0.980812	1.053622	0.985143	1.065367	0.983627	1.073320	0.982791
MPW1PW91	1.192975	0.971334	1.173700	0.974062	1.130356	0.979001	1.141174	0.977674	1.152765	0.976501
O3LYP	1.258053	0.966117	1.244797	0.967998	1.193563	0.973580	1.197151	0.973179	1.213450	0.971588
PBE0	1.185165	0.972432	1.169135	0.974791	1.128500	0.979454	1.135892	0.978556	1.147623	0.977360
X3LYP	1.245282	0.966891	1.232048	0.968775	1.174289	0.974972	1.184339	0.973728	1.198812	0.972285
M05	1.185122	0.972587	1.158272	0.976307	1.119163	0.980843	1.129507	0.979328	1.137780	0.978564
M05–2X	1.173712	0.971929	1.152179	0.974716	1.109623	0.979617	1.126140	0.977497	1.134379	0.976687
M06	1.183815	0.973692	1.158286	0.977109	1.103520	0.983255	1.116857	0.981438	1.126418	0.980535
M06–2X	1.157223	0.974574	1.138148	0.977087	1.096624	0.982006	1.106498	0.980692	1.115064	0.979824
M08-HX	1.162726	0.974969	1.147182	0.977124	1.104384	0.982133	1.127512	0.979073	1.136172	0.978272
M08-SO	1.173555	0.974266	1.147342	0.977998	1.110797	0.982175	1.124917	0.980159	1.133020	0.979436
τ-HCTHh	1.256314	0.966677	1.238518	0.969063	1.186103	0.974663	1.197765	0.973308	1.210254	0.972078
TPSS0	1.204020	0.968892	1.179614	0.972234	1.137001	0.977084	1.148804	0.975677	1.159847	0.974542
CAM-B3LYP	1.166226	0.974034	1.149228	0.976474	1.102029	0.981902	1.111555	0.980645	1.124541	0.979278
HSE-HJS	1.188768	0.972111	1.173426	0.974382	1.130861	0.979248	1.138576	0.978313	1.150682	0.977077
LC-rVV10	1.090525	0.980289	1.070696	0.983307	1.043224	0.986823	1.049539	0.986003	1.060046	0.984873
ωB97X-D	1.158703	0.974917	1.141550	0.977473	1.096316	0.982791	1.114660	0.980355	1.124608	0.979336
ωB97X-D3	1.148885	0.975716	1.130769	0.978407	1.088570	0.983439	1.104463	0.981292	1.114544	0.980255
ωB97X-V	1.145221	0.976150	1.127325	0.978788	1.087580	0.983559	1.095725	0.982474	1.107852	0.981188

a MP2 problem cases removed.

b ClCCCl and HCCCCH removed.

**Table 3:** Power-Law Scaling Errors for Scaled Fundamental Vibrations (in cm^–1^)

method	6–31G(d)			6–31+G(d,p)			6–31+G(2df,p)			6–311+G(d,p)			6–311+G(2df,p)		
	RMSD	MAD	MED	RMSD	MAD	MED	RMSD	MAD	MED	RMSD	MAD	MED	RMSD	MAD	MED
HF	46.2	33.0	22.0	45.1	31.5	19.9	46.3	32.6	19.7	47.1	32.4	19.2	47.3	33.4	20.3
MP2^a^	39.1	24.4	15.6	38.9	26.0	18.0	32.3	19.7	10.7	34.8	21.7	14.2	29.5	17.4	9.7
CCSD^b^	28.4	20.3	15.4	23.0	17.0	12.5	17.0	11.7	8.0	20.4	15.1	11.3	17.7	12.1	8.0
BLYP	37.6	25.8	18.8	29.4	21.9	17.5	25.4	18.7	14.3	30.7	23.0	18.5	26.3	19.7	15.9
BP86	30.0	20.2	13.7	22.5	16.0	11.4	21.1	15.4	11.0	23.2	16.3	11.5	18.9	13.9	10.4
EDF1	29.9	20.5	14.9	23.0	16.6	12.4	21.5	15.4	10.6	23.7	16.8	11.9	20.5	14.5	9.9
HCTH-93	27.9	19.9	14.2	22.6	16.2	11.6	22.7	16.1	11.2	23.8	16.8	11.8	21.5	15.1	9.8
HCTH-120	28.3	19.7	14.1	22.2	15.9	11.5	22.0	15.9	11.4	23.3	16.5	11.7	20.7	14.7	9.8
HCTH-147	28.2	19.7	13.9	22.3	16.0	11.6	22.1	16.0	11.6	23.4	16.6	11.7	20.8	14.8	10.0
HCTH-407	28.7	19.9	13.7	24.4	17.5	12.9	25.4	18.5	13.6	25.4	18.1	13.3	23.7	17.2	12.7
KT3	29.1	19.8	14.4	21.3	15.8	11.9	20.7	15.2	10.8	21.3	15.6	11.7	19.5	14.1	9.9
OLYP	29.3	20.4	14.8	22.9	16.6	12.0	22.5	16.0	10.9	23.7	16.7	11.4	21.5	15.1	10.1
PBE	29.7	19.8	13.5	22.9	16.3	11.5	22.8	17.1	12.7	23.4	16.6	11.6	21.0	15.3	10.8
B97M-V	30.1	21.7	15.4	24.5	17.5	12.6	24.8	17.5	12.9	24.4	17.6	12.8	22.9	16.4	11.9
rSCAN	23.9	16.5	11.6	18.8	13.5	9.5	19.8	15.3	12.7	19.8	14.2	10.2	17.8	13.1	9.9
SCAN	32.4	22.5	15.2	28.9	19.5	13.8	26.2	17.7	12.5	29.2	20.1	14.1	27.6	18.1	11.7
τ-HCTH	28.4	19.8	14.3	21.4	15.4	11.2	20.0	14.4	10.3	22.0	15.5	11.1	19.0	13.5	9.2
TPSS	37.8	25.2	18.2	29.0	21.4	17.6	23.7	17.0	12.8	27.8	20.9	17.1	23.7	17.7	14.0
VSXC	30.1	21.1	15.3	24.1	17.2	12.4	23.4	17.2	13.3	24.9	17.4	12.3	22.4	16.3	12.5
B1LYP	27.9	19.9	15.0	19.7	14.7	11.0	18.0	12.8	8.1	20.5	15.2	11.4	18.0	13.1	9.7
B3LYP	27.7	19.5	14.3	19.5	14.4	10.5	17.8	12.6	8.4	20.4	14.9	10.9	17.6	12.7	9.0
B3P86	25.2	17.5	11.2	19.1	13.7	9.8	20.3	15.2	12.2	19.8	14.3	10.4	18.9	13.9	10.4
B3PW91	25.0	17.4	11.1	19.4	13.8	9.8	20.5	15.2	11.8	20.2	14.5	10.3	19.4	14.0	10.1
B97	24.0	17.0	12.1	16.8	11.8	7.7	16.5	11.8	8.1	18.2	12.7	8.5	16.1	11.3	7.4
B97–K	26.7	19.2	13.4	20.3	14.5	10.2	19.8	13.9	9.4	21.3	15.2	10.2	20.2	14.3	9.8
B97–1	24.0	16.9	11.6	16.8	11.8	8.0	16.6	11.9	8.3	18.1	12.6	8.4	16.2	11.4	7.2
B97–2	24.1	16.7	11.5	19.0	13.5	9.8	20.1	15.0	12.1	20.1	14.3	10.0	19.2	13.9	9.9
B97–3	23.8	17.0	11.6	18.5	13.0	9.1	19.2	13.8	9.8	19.7	13.8	9.3	18.5	13.1	9.3
BHHLYP	30.2	21.4	13.8	25.2	17.7	11.7	25.8	18.4	13.5	26.2	18.4	11.7	25.9	18.4	12.5
EDF2	26.4	18.2	12.0	19.1	13.6	9.7	19.2	14.1	10.4	19.9	14.2	10.0	18.0	12.8	8.9
MPW1K	29.7	20.8	14.3	27.2	19.5	14.7	29.2	21.5	16.5	28.3	20.4	15.5	29.0	21.3	16.1
MPW1PW91	25.5	17.7	11.0	20.6	14.9	10.8	22.3	16.6	12.9	21.5	15.7	11.4	21.4	15.7	11.7
O3LYP	25.9	18.0	12.2	20.0	14.3	10.0	20.8	14.9	10.6	21.1	15.0	10.5	19.9	14.0	9.4
PBE0	26.3	18.2	11.3	22.0	16.2	12.0	24.2	18.4	14.9	22.9	17.0	13.0	23.1	17.3	13.6
X3LYP	27.5	19.3	14.2	19.3	14.1	10.4	17.9	12.7	8.5	20.1	14.6	10.5	17.5	12.6	8.6
M05	27.3	19.0	13.4	24.9	18.8	15.6	27.3	21.2	18.6	26.1	19.4	15.0	26.3	20.0	16.7
M05–2X	30.5	20.1	12.5	25.7	17.5	11.9	26.5	18.8	13.9	24.5	16.8	11.7	24.5	17.0	11.7
M06	30.0	21.7	15.7	26.6	20.0	15.8	29.2	22.5	19.0	27.7	20.7	16.5	27.3	20.6	16.8
M06–2X	30.2	20.4	13.4	25.1	17.6	12.9	26.6	19.4	15.4	24.5	17.3	12.5	25.1	18.0	14.3
M08-HX	33.3	23.3	15.0	30.4	21.9	16.2	32.5	23.9	18.8	30.5	22.0	16.3	31.2	22.6	17.0
M08-SO	31.4	22.3	16.0	27.4	20.0	15.9	29.1	21.5	17.4	28.3	20.8	16.3	28.7	21.2	17.2
τ-HCTHh	24.1	16.8	11.6	16.9	11.9	8.0	16.6	12.0	8.6	18.1	12.7	8.7	15.7	11.1	7.1
TPSS0	28.3	19.5	13.7	20.0	14.3	10.4	18.4	12.9	8.9	19.6	14.1	10.2	18.4	13.2	9.4
CAM-B3LYP	27.9	18.8	11.6	21.4	15.2	10.6	21.6	15.6	11.0	21.5	15.3	10.6	20.5	14.5	10.3
HSE-HJS	26.2	18.1	11.2	21.7	15.9	11.7	23.8	18.1	14.6	22.6	16.7	12.6	22.7	16.9	13.4
LC-rVV10	34.4	24.9	18.9	33.0	24.7	19.4	34.6	26.8	22.4	33.1	25.0	20.2	33.5	25.6	20.7
ωB97X-D	25.2	18.0	12.0	21.5	15.8	11.3	22.5	16.5	12.4	22.4	16.2	11.4	21.7	15.9	11.5
ωB97X-D3	25.7	18.1	12.2	22.4	16.4	12.3	23.7	17.8	14.5	23.1	16.8	12.4	22.7	16.8	13.0
ωB97X-V	27.4	18.9	12.6	22.8	16.5	12.3	23.2	17.5	14.3	22.7	16.6	12.4	22.3	16.4	12.8

a MP2 problem cases removed.

b ClCCCl and HCCCCH removed.

**Table 4:** Power-Law Scaling Errors for Scaled Fundamental Vibrations (in cm^–1^) (cont.)

method	6–31++G(d,p)			6–311++G(d,p)			6–311++G(2df,p)			cc-pVTZ			aug-cc-pVTZ		
	RMSD	MAD	MED	RMSD	MAD	MED	RMSD	MAD	MED	RMSD	MAD	MED	RMSD	MAD	MED
HF	45.1	31.4	20.0	47.1	32.4	19.0	47.4	33.4	20.0	48.3	34.2	21.0	46.9	33.3	20.3
MP2^a^	39.1	26.4	18.1	35.5	22.4	14.4	29.4	17.3	9.8	30.0	17.2	9.6	31.4	18.8	10.8
CCSD^b^	23.6	17.3	12.7	46.8	19.4	13.6	17.7	12.0	8.0	18.9	13.1	8.8			
BLYP	29.5	21.8	17.5	30.6	23.0	18.5	26.2	19.6	15.8	26.8	20.2	17.0	28.2	21.0	17.3
BP86	22.5	16.0	11.4	23.1	16.3	11.4	20.0	14.3	9.9	20.6	14.8	10.1	21.2	15.1	10.4
EDF1	23.0	16.6	12.4	23.7	16.8	11.9	20.5	14.5	9.9	20.7	14.8	10.1	21.4	15.3	10.3
HCTH-93	22.6	16.2	11.7	23.8	16.8	11.9	21.5	15.2	10.0	21.5	15.1	10.1	21.6	15.2	10.4
HCTH-120	22.2	15.9	11.3	23.2	16.4	11.6	20.7	14.7	9.8	20.8	14.8	10.2	21.1	15.0	10.3
HCTH-147	22.3	16.1	11.4	23.3	16.6	11.6	20.8	14.8	10.1	20.9	15.0	10.1	21.2	15.1	10.3
HCTH-407	24.5	17.6	12.8	25.4	18.1	13.3	23.8	17.3	12.8	23.7	17.1	12.5	23.7	17.0	12.1
KT3	21.3	15.8	11.9	21.3	15.6	11.5	19.5	14.1	10.0	19.5	14.2	10.4	19.9	14.4	10.5
OLYP	22.9	16.7	11.9	23.7	16.7	11.3	21.5	15.2	10.1	21.2	14.9	10.0	21.3	15.0	10.1
PBE	22.9	16.4	11.6	23.4	16.6	11.6	21.1	15.3	10.9	21.4	15.6	11.2	21.8	15.7	10.9
B97M-V	24.6	17.5	12.7	24.3	17.6	13.1	23.0	16.4	12.0	23.6	16.5	12.1	23.1	16.4	12.0
rSCAN	18.8	13.6	9.7	19.8	14.2	10.2	17.8	13.1	10.0	17.9	13.1	9.8	18.0	13.2	9.8
SCAN	29.3	19.5	13.8	29.7	20.1	13.9	27.3	17.8	11.7	28.8	18.4	12.3	28.2	18.0	12.1
τ-HCTH	21.4	15.3	11.0	22.0	15.5	11.0	19.1	13.5	9.2	19.7	14.1	9.8	19.8	14.2	9.5
TPSS	29.0	21.3	17.5	27.7	20.8	17.0	23.6	17.7	13.8	24.2	18.0	14.0	25.0	18.6	14.7
VSXC	24.2	17.4	12.4	24.9	17.4	12.3	22.4	16.3	12.6	21.8	15.9	12.3	22.5	16.4	12.5
B1LYP	19.7	14.7	11.1	20.5	15.2	11.2	18.0	13.1	9.6	19.1	14.1	10.4	19.0	14.1	10.5
B3LYP	19.5	14.4	10.5	20.3	14.9	10.9	17.6	12.7	8.9	18.5	13.6	10.0	18.6	13.7	10.2
B3P86	19.2	13.7	9.9	19.8	14.3	10.4	18.9	13.9	10.4	19.6	14.2	10.1	19.0	13.7	9.7
B3PW91	19.4	13.8	9.7	20.2	14.5	10.3	18.9	13.9	10.4	19.9	14.3	10.0	19.4	13.8	9.5
B97	16.8	11.8	7.7	18.2	12.7	8.6	16.2	11.4	7.4	16.9	12.1	8.2	16.3	11.7	7.7
B97–K	20.3	14.5	10.0	21.3	15.2	10.1	20.2	14.4	9.9	21.4	15.4	10.8	20.6	14.9	10.1
B97–1	16.8	11.7	8.0	18.1	12.6	8.4	16.2	11.4	7.3	17.1	12.2	8.3	16.4	11.8	8.1
B97–2	19.0	13.5	10.0	20.1	14.3	10.1	19.3	13.9	10.0	19.7	14.2	9.9	19.0	13.6	9.6
B97–3	18.5	13.1	9.1	19.7	13.8	9.3	18.6	13.2	9.3	19.0	13.6	9.6	18.2	13.0	9.2
BHHLYP	25.2	17.7	11.6	26.2	18.3	11.5	25.9	18.4	12.6	26.8	18.9	13.1	25.6	18.1	12.5
EDF2	19.2	13.6	9.6	19.9	14.2	9.9	18.0	12.8	8.9	18.9	13.5	9.6	18.5	13.2	9.2
MPW1K	27.1	19.5	14.6	28.3	20.4	15.5	29.1	21.3	16.0	29.6	21.7	16.4	28.3	20.7	15.7
MPW1PW91	20.6	14.9	10.7	21.5	15.7	11.6	21.4	15.7	12.0	21.9	15.9	11.4	21.0	15.3	11.2
O3LYP	20.0	14.3	10.2	21.1	15.0	10.5	20.0	14.1	9.6	20.2	14.2	9.9	19.7	13.7	9.5
PBE0	22.0	16.2	11.9	22.9	17.0	13.3	23.1	17.3	13.5	23.7	17.5	13.0	22.7	16.8	12.9
X3LYP	19.3	14.1	10.4	20.0	14.5	10.4	17.5	12.5	8.5	18.6	13.5	9.5	18.4	13.4	9.7
M05	24.9	18.8	15.8	26.2	19.4	15.1	26.4	20.0	16.9	26.4	20.3	16.9	25.3	19.5	16.7
M05–2X	25.8	17.6	12.1	24.4	16.7	11.6	24.5	17.0	11.9	24.9	17.4	12.0	24.5	17.1	11.7
M06	26.7	20.1	15.9	27.7	20.7	16.6	27.4	20.7	17.0	27.7	20.3	15.1	26.8	19.8	15.1
M06–2X	25.2	17.7	12.9	24.5	17.3	12.7	25.1	18.0	14.3	24.9	17.9	14.2	24.1	17.2	13.3
M08-HX	30.4	21.9	16.2	30.6	22.1	16.3	31.3	22.7	17.1	30.3	22.0	16.4	29.7	21.7	16.4
M08-SO	27.5	20.0	16.0	28.3	20.8	16.2	28.7	21.2	17.1	28.3	20.6	16.1	27.8	20.3	15.8
τ-HCTHh	16.9	11.9	7.9	18.1	12.7	8.8	15.8	11.1	7.3	16.5	11.6	7.5	16.1	11.4	7.4
TPSS0	20.0	14.3	10.4	19.6	14.1	10.1	18.4	13.1	9.3	19.6	14.1	10.0	19.0	13.7	10.1
CAM-B3LYP	21.4	15.2	10.6	21.5	15.3	10.8	20.5	14.5	10.4	21.3	15.1	10.7	20.7	14.7	10.4
HSE-HJS	21.7	15.9	11.7	22.7	16.7	12.8	22.8	17.0	13.4	23.4	17.2	12.8	22.4	16.5	12.6
LC-rVV10	33.0	24.7	19.3	33.1	25.0	20.1	33.5	25.6	20.8	33.7	25.6	21.2	32.9	24.9	19.9
ωB97X-D	21.5	15.7	11.2	22.4	16.2	11.5	21.8	15.9	11.6	21.5	15.8	11.5	20.8	15.3	11.2
ωB97X-D3	22.4	16.4	12.3	23.1	16.7	12.5	22.8	16.9	13.0	22.4	16.6	12.6	21.9	16.2	12.0
ωB97X-V	22.8	16.5	12.3	22.7	16.6	12.3	22.3	16.4	12.8	22.7	16.6	13.0	22.0	16.1	12.4

a MP2 problem cases removed.

b ClCCCl and HCCCCH removed.

The B97/HCTH functionals have similar functional forms and can be considered the best-performing family of functionals for predicting these vibrational properties. B97–1 is known to be relatively accurate for calculating anharmonic vibrational frequencies,^6,135,136^ and has previously been recommended alongside the ωB97X-D and TPSS0 functional forms for uniform scaling calculations.^19,22^ However, while the range-separated ωB97X-D and TPSS0 functionals both perform well for uniform scaling, they are not improved to the same extent by WPS, with their 6–311++G­(2df,p) RMSDs dropping from 25.7 and 22.0 cm^–1^ to just 21.8 and 18.4 cm^–1^, respectively. This suggests that the success of ωB97X-D and TPSS0 may be due to error cancellation in the uniform scaling factors. The Minnesota family of meta-hybrid functionals (e.g., M06, M06–2X, etc.) are known to be less accurate for calculating both anharmonic and uniformly scaled frequencies,^26,41,136^ and while they improve in absolute terms when using WPS here, they also improve to a lesser degree than other methods. For example, the M06–2X/6–311++G­(2df,p) RMSD falls from 28.6 to 25.1 cm^–1^ when using WPS instead of uniform scaling.

DFT GGA functionals are less computationally intensive to evaluate than hybrid or meta-GGA functionals but tend to produce harmonic frequencies that are resistant to uniform scaling with scaling factors close to unity. In contrast, the GGA functionals tested here are consistently improved by power law scaling. The BLYP/6–311++G­(2df,p) RMSD drops from 37.6 to 26.2 cm^–1^ when using WPS instead of uniform scaling, and the KT3 functional is found to be the best-performing GGA with a 19.5 cm^–1^ WPS RMSD using the same basis set. The WPS KT3 frequencies also outperform many uniformly scaled frequencies calculated with hybrid and meta-hybrid functionals.

The least accurate method tested here is Hartree–Fock theory, as the harmonic deviations are approximately uniform and are not improved by WPS. Instead, the HF power law converges toward uniform scaling, with the power approaching unity, e.g., for HF/6–311++G­(2df,p) λ_2_ = 0.997854. WPS HF frequencies are then progressively improved by adding MP2 and CCSD correlation corrections, indicating that power law scaling does not meaningfully compensate for the neglect of electron correlation.

### Basis-Set Testing

Scaling factors have also been determined using additional basis sets for some of the most promising methods (see Tables 5–6 and the Supporting Information). The best-performing method combinations include the Def2-TZVPD basis set, which gives WPS RMSDs of 15.3, 15.3, and 15.4 cm^–1^ for the B97, B97–1, and τ-HCTHh functionals, respectively. The HCTHh functional performs slightly worse than B97 and B97–1 in this case, while the B97/Def2-TZVPD WPS improves on uniform scaling by ca. 40% in RMSD and ca. 64% in MED. Def2-TZVPD B97, B97–1, and the τ-HCTHh calculations are therefore recommended to users here for the practical calculation of vibrational properties, with B97 and B97–1 recommended for their combination of speed and accuracy, and τ-HCTHh recommended in cases where explicit dispersion and complementary meta-hybrid properties are important.

**Table 5:** Power-Law Scaling Factors for Fundamental Vibrations with Additional Basis Sets

method	B3LYP		B97		B97–1		τ-HCTHh	
	λ_1_	λ_2_	λ_1_	λ_2_	λ_1_	λ_2_	λ_1_	λ_2_
6–31G(d)	1.217240	0.969138	1.206444	0.970536	1.201484	0.970893	1.216724	0.969966
6–31+G(d,p)	1.258469	0.965749	1.244420	0.967410	1.238242	0.967891	1.256712	0.966611
6–31++G(d,p)	1.258020	0.965817	1.244086	0.967470	1.237797	0.967963	1.256314	0.966677
6–31+G(2df,p)	1.211171	0.971212	1.204829	0.972121	1.198939	0.972585	1.213310	0.971666
6–311+G(d,p)	1.245463	0.967594	1.228419	0.969642	1.222080	0.970145	1.237626	0.969143
6–311++G(d,p)	1.246466	0.967500	1.229379	0.969553	1.223094	0.970048	1.238518	0.969063
6–311+G(2df,p)	1.187153	0.973779	1.178036	0.975098	1.172511	0.975524	1.186178	0.974641
6–311++G(2df,p)	1.187106	0.973795	1.178034	0.975111	1.172534	0.975533	1.186103	0.974663
6–311++G(3df,3pd)	1.180797	0.974337	1.172760	0.975583	1.167361	0.975996	1.180113	0.975187
pc-2	1.183629	0.974044	1.171319	0.975757	1.166439	0.976104	1.180802	0.975082
pc-3	1.175915	0.974956	1.167389	0.976271	1.162202	0.976654	1.176018	0.975691
aug-pc-2	1.191452	0.973234	1.179502	0.974901	1.174275	0.975286	1.189908	0.974131
aug-pc-3	1.176366	0.974908	1.168205	0.976183	1.162889	0.976580	1.177297	0.975551
Def2-TZVP	1.176560	0.974839	1.169756	0.975944	1.164735	0.976308	1.179961	0.975217
Def2-TZVPD	1.184572	0.974029	1.177216	0.975198	1.171881	0.975591	1.186944	0.974528
cc-pVDZ	1.259194	0.966571	1.252639	0.967432	1.247594	0.967800	1.263715	0.966802
cc-pVTZ	1.197227	0.972559	1.186771	0.974107	1.181607	0.974486	1.197765	0.973308
cc-pVQZ	1.184078	0.974054	1.176087	0.975301	1.171373	0.975625	1.185250	0.974689
aug-cc-pVDZ	1.315435	0.960959	1.301029	0.962646	1.295435	0.963043	1.313221	0.961934
aug-cc-pVTZ	1.211845	0.971112	1.200326	0.972766	1.194689	0.973189	1.210254	0.972078
aug-cc-pVQZ	1.188230	0.973630	1.180183	0.974884	1.175040	0.975253	1.189393	0.974267

**Table 6:** Power-Law Scaling Errors for Fundamental Vibrations (in cm^–1^) with Additional Basis Sets

method	B3LYP			B97			B97–1			τ-HCTHh		
	RMSD	MAD	MED	RMSD	MAD	MED	RMSD	MAD	MED	RMSD	MAD	MED
6–31G(d)	27.7	19.5	14.3	24.0	17.0	12.1	24.0	16.9	11.6	24.1	16.8	11.6
6–31+G(d,p)	19.5	14.4	10.5	16.8	11.8	7.7	16.8	11.8	8.0	16.9	11.9	8.0
6–31++G(d,p)	19.5	14.4	10.5	16.8	11.8	7.7	16.8	11.7	8.0	16.9	11.9	7.9
6–31+G(2df,p)	17.8	12.6	8.4	16.5	11.8	8.1	16.6	11.9	8.3	16.6	12.0	8.6
6–311+G(d,p)	20.4	14.9	10.9	18.2	12.7	8.5	18.1	12.6	8.4	18.1	12.7	8.7
6–311++G(d,p)	20.3	14.9	10.9	18.2	12.7	8.6	18.1	12.6	8.4	18.1	12.7	8.8
6–311+G(2df,p)	17.6	12.7	9.0	16.1	11.3	7.4	16.2	11.4	7.2	15.7	11.1	7.1
6–311++G(2df,p)	17.6	12.7	8.9	16.2	11.4	7.4	16.2	11.4	7.3	15.8	11.1	7.3
6–311++G(3df,3pd)	17.5	12.5	8.4	16.1	11.3	7.2	16.2	11.4	7.3	16.0	11.2	7.4
pc-2	17.5	12.4	8.3	15.8	11.0	6.9	15.8	11.0	7.1	15.8	11.0	7.1
pc-3	17.4	12.4	8.5	15.8	11.0	6.9	15.9	11.0	7.0	15.6	10.8	6.9
aug-pc-2	17.7	12.6	8.5	15.8	11.0	7.0	15.8	11.0	7.0	15.7	11.0	7.0
aug-pc-3	17.4	12.4	8.4	15.8	11.0	6.9	15.9	11.0	6.9	15.6	10.8	6.9
Def2-TZVP	17.3	12.4	8.5	15.3	10.6	7.0	15.3	10.7	7.0	15.4	10.7	7.2
Def2-TZVPD	17.5	12.5	8.4	15.3	10.5	6.9	15.3	10.6	7.0	15.4	10.7	7.2
cc-pVDZ	24.6	17.7	12.9	21.8	16.5	12.9	21.9	16.5	12.8	22.5	17.1	13.7
cc-pVTZ	18.5	13.6	10.0	16.9	12.1	8.2	17.1	12.2	8.3	16.5	11.6	7.5
cc-pVQZ	17.6	12.8	9.1	16.1	11.3	7.4	16.1	11.3	7.4	15.8	11.1	7.2
aug-cc-pVDZ	22.7	16.5	12.2	20.5	14.9	11.4	20.5	15.0	11.2	21.2	15.6	11.6
aug-cc-pVTZ	18.6	13.7	10.2	16.3	11.7	7.7	16.4	11.8	8.1	16.1	11.4	7.4
aug-cc-pVQZ	17.8	12.9	9.3	16.0	11.2	7.2	16.0	11.2	7.3	15.8	11.1	7.1

WPS frequencies are found to be generally less sensitive to basis-set completeness than the uniformly scaled frequencies and converge more rapidly with respect to the basis-set size. For example, B97/6–31+G­(d,p) has a WPS RMSD just ca. 0.7 cm^–1^ higher than the largest Pople basis set, 6–311++G­(3df,3pd), and ca. 0.8 cm^–1^ higher than the quadruple-ζ aug-cc-pVQZ basis set. By comparison, uniform scaling factors give RMSDs that are ca. 3.6 and 3.9 cm^–1^ higher for these same methods. These basis-set trends are shown in Figure 2, and indicate that WPS frequencies can be both more accurate and more computationally efficient to calculate.

**Figure 2:**
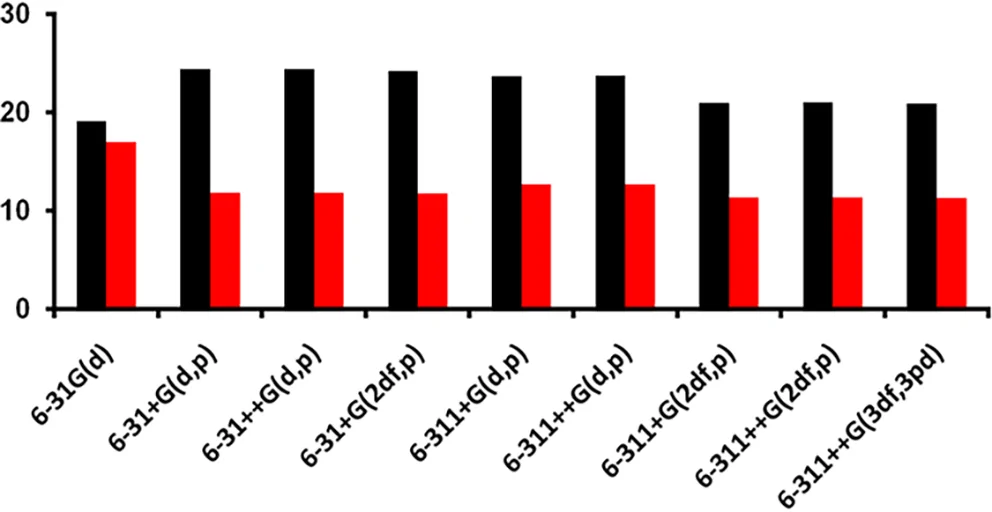
Uniform scaling (black) and power law scaling (red) MADs (in cm^–1^) for the Pople basis sets with the B97 functional.

The smallest basis set tested here is 6–31G­(d), which does not include either diffuse basis functions or polarization functions on hydrogen atoms. Systematic cancellation of errors in the uniform scaling factors make the 6–31G­(d) basis set particularly effective for uniform scaling. However, this is no longer the case for WPS. Instead, the “ideal scaling factors” (*ν_i_*/*ω_i_*) increase over the higher energy bond stretching region. Improvements when using WPS are therefore less consistent for this basis set, and WPS frequencies should be considered particularly sensitive to these kinds of basis set and method imbalances. However, the WPS 6–31G­(d) frequencies still improve on the uniformly scaled frequencies for the best-performing methods, indicating that power law scaling may be better able to compensate for these basis-set deficiencies than for large errors in electron correlation.

### Thermodynamic Properties

The fundamental vibrational transition frequencies also determine the thermal vibrational enthalpy and entropy corrections in eqs 6 and 7. The objective functions that determine the optimal scaling factors for each property differ due to the frequencies entering the thermodynamic equations in nonlinear combinations related to the temperature. This has led previous authors to use different scaling factors to minimize the errors in each property at various temperatures.^16,31^ However, the ideal scaling factors for each mode, ν_i_/ω_i_, minimize the residuals for all three properties at any given temperature. Differences between the “optimal” scaling factors for thermodynamic and fundamental vibrational properties are therefore due to limitations in the scaling methods that prevent them from perfectly reproducing these ideal factors. Given the strong performance of WPS compared to uniform scaling, specialized thermodynamic scaling factors may be unnecessary. To test this, thermal enthalpy and entropy contributions have been calculated using both scaling factors optimized for these thermodynamic contributions, and the scaling factors previously optimized for the fundamental transition frequencies. The results are summarized in Table 7, with the additional scaling factors provided in the Supporting Information.

**Table 7:** Scaled Vibrational Thermal Enthalpy Δ*H*_vib_(*T*) and Entropy *S*_vib_(*T*) Contribution RMSDs Calculated at 298.15 K with Scaling Factors Optimized for Fundamentals (λ_ω_), Enthalpies (λ_H_), and Entropies (λ_S_), and Their Absolute Differences (Δλ)

	Δ*H*_vib_(*T*) (kJ/mol)						*S*_vib_(*T*) (J/mol/K)					
method	uniform scaling			power scaling			uniform scaling			power scaling		
	λ_ω_	λ_H_	Δλ	λ_ω_	λ_H_	Δλ	λ_ω_	λ_S_	Δλ	λ_ω_	λ_S_	Δλ
B3LYP/6–31G(d)	0.028	0.017	0.011	0.015	0.014	5 × 10^–4^	0.18	0.10	0.08	0.09	0.09	3 × 10^–3^
B97/6–31G(d)	0.027	0.016	0.011	0.014	0.013	4 × 10^–4^	0.17	0.09	0.08	0.08	0.08	2 × 10^–3^
B97–1/6–31G(d)	0.027	0.016	0.011	0.014	0.013	4 × 10^–4^	0.17	0.09	0.08	0.08	0.08	2 × 10^–3^
τ-HCTHh/6–31G(d)	0.028	0.016	0.011	0.014	0.014	4 × 10^–4^	0.18	0.10	0.08	0.09	0.09	2 × 10^–3^
B3LYP/Def2-TZVPD	0.022	0.012	0.010	0.010	0.010	8 × 10^–6^	0.14	0.07	0.07	0.06	0.06	3 × 10^–4^
B97/Def2-TZVPD	0.020	0.011	0.010	0.009	0.009	7 × 10^–6^	0.13	0.07	0.07	0.05	0.05	1 × 10^–4^
B97–1/Def2-TZVPD	0.020	0.011	0.009	0.009	0.009	2 × 10^–5^	0.13	0.07	0.07	0.05	0.05	2 × 10^–4^
τ-HCTHh/Def2-TZVPD	0.021	0.011	0.010	0.009	0.009	1 × 10^–5^	0.14	0.07	0.07	0.06	0.06	1 × 10^–4^

Optimizing the uniform scaling factors for thermal enthalpy and entropy contributions reduces the enthalpy RMSD by around 0.010 kJ/mol and reduces the entropy RMSD by around 0.07 to 0.08 J/mol/K RMSD. This corresponds to a ca. 47% improvement in the enthalpies when using B97/Def2-TZVPD (from 0.020 to 0.011 kJ/mol RMSD) and a ca. 51% improvement in the entropies (from 0.13 to 0.07 J/mol/K RMSD). In contrast, the power law scaling factors generate superior predictions for both properties without property specific optimizations, giving an enthalpy RMSD of 0.009 kJ/mol and an entropy RMSD of 0.05 J/mol/K. Further optimizing the power law factors for the enthalpy and entropy contributions specifically, only improves these RMSDs to a minor degree. The enthalpies are improved by between ca. 5 × 10^–4^ and 7 × 10^–6^ kJ/mol and the entropies are improved by between 3 × 10^–3^ and 1 × 10^–4^ J/mol/K. This corresponds to ca. 0.07 and 0.13% improvements for B97/Def2-TZVPD. Power scaling factors are therefore able to treat fundamental frequencies, vibrational enthalpies, and vibrational entropies using a single consistent set of scaling factors.

### Large Molecule Tests

Scaled fundamental frequencies have also been calculated for the naphthalene and benzonitrile molecules to test how these WPS factors perform for larger molecules that are outside of the parametrization set. Both molecules are significantly larger than the molecules in the F1 set, and both molecules have delocalized aromatic π-systems that are also absent from the set. Scaled fundamental frequencies are shown for these molecules in Tables 8 and 9. Benzonitrile gives a WPS RMSD ca. 34% lower than uniform scaling for the τ-HCTHh/Def2-TZVPD method. However, modes 1, 2, 3, 6, and 7 of benzonitrile also contain significant amounts of C≡N triple bond bending character. With these modes removed, the WPS RMSD improves further to become ca. 40% better than uniform scaling across the remaining 28 modes. Naphthalene is an even more significant departure from the F1 set, with an extended aromatic system delocalized over two benzenoid rings and a relatively constrained carbon framework that further restricts how some of the low frequency bending modes can move. Consequently, uniform scaling factors outperform WPS in the low frequency region below 1000 cm^–1^, while WPS outperforms uniform scaling in the bond stretching modes above 1000 cm^–1^. The result is that WPS gives improved MAD and MED values, but similar RMSDs for the B97 and B97–1 functionals. However, the ninth bending mode is found to be particularly inaccurate for both uniform scaling and WPS. Uniform scaling factors give errors of 28–29 cm^–1^ for this mode, and WPS factors give errors of 48–50 cm^–1^. With mode 9 removed, the WPS frequencies again consistently improve on the uniform scaled frequencies across all three accuracy measures. These larger molecules therefore indicate that WPS can improve the calculated frequencies of molecules with bonding and vibrational types that are significantly outside of the F1 set in terms of both size and bond structure. However, they also highlight the need to carefully identify the modes that deviate from these general trends. The exclusion of anomalous and triple bond bending modes from the fitting data can make these scaling factors more accurate overall but also make them less reliable for these types of vibrational mode, with similar effects seen here for modes involving structural constraints on bending. Users should therefore be mindful of these limitations when applying WPS factors.

**Table 8:** Uniform and Power-Law Scaled Frequencies and Accuracies for the Fundamental Vibrations of Benzonitrile (in cm^–1^) Calculated Using the Def2-TZVPD Basis Set

	uniform scaling			power scaling		
exp.^137^	B97	B97–1	τ-HCTHh	B97	B97–1	τ-HCTHh
141	139.69	139.89	139.13	149.82	149.85	149.57
167	160.24	159.85	159.29	171.28	170.67	170.64
372	377.16	377.17	376.34	394.66	394.34	394.43
399	390.74	391.21	389.36	408.51	408.66	407.73
462	447.92	447.85	448.13	466.70	466.29	467.59
542	546.15	545.64	545.20	566.27	565.38	566.04
549	547.68	547.41	545.83	567.81	567.16	566.68
620	614.81	614.65	614.86	635.58	635.03	636.41
688	681.97	681.99	679.76	703.21	702.82	701.79
756	744.25	744.24	744.88	765.77	765.33	767.23
762	751.19	751.17	747.93	772.73	772.29	770.30
845	832.17	832.10	826.23	853.85	853.36	848.78
922	915.89	915.81	910.65	937.53	937.01	933.19
975	961.97	962.13	956.31	983.50	983.22	978.76
990	981.79	981.96	979.63	1003.26	1002.98	1002.01
1002	984.14	984.05	981.68	1005.60	1005.06	1004.06
1029	1012.52	1012.26	1011.36	1033.86	1033.16	1033.63
1076	1065.93	1065.24	1064.22	1087.02	1085.89	1086.24
1154	1144.99	1143.86	1142.81	1165.57	1164.01	1164.34
1179	1158.24	1157.37	1154.90	1178.72	1177.42	1176.35
1193	1176.02	1175.97	1176.63	1196.36	1195.88	1197.91
1287	1278.67	1277.66	1282.57	1298.10	1296.66	1302.90
1378	1308.65	1307.92	1308.85	1327.76	1326.61	1328.91
1456	1425.82	1425.74	1424.19	1443.57	1443.07	1442.91
1496	1470.65	1470.51	1468.09	1487.82	1487.26	1486.24
1591	1560.39	1560.67	1559.80	1576.28	1576.16	1576.65
1602	1584.79	1584.95	1584.16	1600.32	1600.08	1600.64
2242	2252.33	2254.64	2250.11	2254.66	2256.65	2253.29
3030	3073.84	3073.34	3076.64	3053.38	3052.92	3056.52
3033	3083.60	3082.98	3085.95	3062.83	3062.26	3065.54
3051	3091.83	3090.97	3094.26	3070.80	3070.01	3073.58
3081	3100.21	3099.13	3102.16	3078.92	3077.91	3081.23
3087	3103.22	3102.24	3105.34	3081.84	3080.92	3084.31
MAD	17.2	17.3	18.7	12.6	12.4	12.3
MED	12.8	12.9	14.1	11.0	9.9	10.3
RMSD	22.8	22.8	24.1	15.9	15.8	15.9
MAD^a^	19.6	19.7	21.3	12.1	11.9	11.8
MED^a^	15.2	14.7	17.7	10.9	9.8	10.3
RMSD^a^	24.7	24.7	26.1	15.6	15.5	15.7

a Modes 1, 2, 3, 6, and 7 were removed.

**Table 9:** Uniform and Power-Law Scaled Frequencies and Accuracies for the Fundamental Vibrations of Naphthalene (in cm^–1^) Calculated Using the Def2-TZVPD Basis Set

	uniform scaling			power scaling		
exp.^138^	B97	B97–1	τ-HCTHh	B97	B97–1	τ-HCTHh
176	168.67	168.46	168.19	180.05	179.63	179.93
195	177.73	178.28	176.71	189.48	189.84	188.81
359	350.92	350.35	351.23	367.86	366.97	368.76
386	383.89	384.39	383.99	401.53	401.72	402.24
461	460.43	460.90	459.68	479.42	479.54	479.33
476	473.85	473.87	473.79	493.04	492.70	493.67
506	497.13	497.05	497.23	516.65	516.20	517.45
512	500.47	500.37	500.74	520.03	519.57	521.01
581	609.46	609.14	609.73	630.19	629.48	631.24
618	615.20	615.99	615.42	635.99	636.38	636.98
717	705.10	705.25	701.60	726.45	726.20	723.75
753	746.61	746.65	746.45	768.13	767.75	768.81
758	762.60	764.21	764.15	784.17	785.36	786.57
770	774.26	774.30	770.55	795.87	795.48	792.99
782	779.21	779.12	779.53	800.83	800.31	801.99
841	822.33	822.67	819.99	844.01	843.92	842.53
876	870.47	871.18	868.49	892.16	892.44	891.06
935	918.27	918.32	918.08	939.90	939.52	940.61
943	934.20	934.62	930.39	955.80	955.78	952.89
958	953.96	954.29	950.65	975.51	975.40	973.12
970	964.73	965.10	960.36	986.25	986.17	982.80
980	970.28	970.73	966.29	991.78	991.79	988.71
1008	1000.46	999.82	1001.09	1021.86	1020.78	1023.39
1025	1010.80	1009.90	1010.81	1032.15	1030.82	1033.08
1125	1110.61	1110.03	1108.90	1131.43	1130.40	1130.66
1138	1128.51	1126.93	1128.05	1149.21	1147.19	1149.68
1145	1130.93	1130.25	1128.35	1151.61	1150.49	1149.98
1158	1142.44	1141.30	1138.96	1163.03	1161.47	1160.52
1209	1190.14	1189.65	1191.54	1210.37	1209.44	1212.70
1239	1225.30	1224.78	1224.08	1245.22	1244.27	1244.96
1265	1243.00	1242.55	1242.26	1262.77	1261.88	1262.98
1361	1346.11	1344.70	1350.35	1364.82	1362.99	1369.95
1376	1352.35	1352.37	1355.99	1370.99	1370.57	1375.53
1389	1369.68	1369.37	1367.57	1388.12	1387.38	1386.98
1438	1436.75	1436.69	1435.37	1454.37	1453.88	1453.95
1460	1440.98	1440.37	1439.78	1458.53	1457.51	1458.30
1506	1494.29	1493.99	1494.39	1511.13	1510.43	1512.18
1577	1557.12	1557.32	1556.73	1573.06	1572.85	1573.62
1595	1582.67	1582.57	1581.57	1598.23	1597.73	1598.09
1624	1610.30	1610.65	1610.31	1625.44	1625.38	1626.38
3027	3059.06	3058.23	3061.42	3039.06	3038.28	3041.79
3031	3060.26	3059.32	3062.39	3040.22	3039.33	3042.73
3058	3063.29	3062.25	3065.56	3043.16	3042.17	3045.80
3060	3065.92	3064.61	3067.82	3045.71	3044.45	3047.98
3060	3076.84	3076.07	3079.01	3056.28	3055.56	3058.82
3065	3077.81	3076.84	3079.80	3057.22	3056.32	3059.58
3090	3089.23	3088.49	3091.30	3068.28	3067.60	3070.72
3092	3090.07	3089.25	3092.11	3069.10	3068.33	3071.50
MAD	11.6	11.7	12.5	11.3	11.1	11.0
MED	11.6	11.7	12.1	9.3	8.9	9.4
RMSD	14.0	14.0	14.8	14.2	14.1	14.0
MAD^a^	11.3	11.3	12.1	10.5	10.3	10.1
MED^a^	11.5	11.6	11.6	9.2	8.7	9.0
RMSD^a^	13.5	13.5	14.4	12.5	12.4	12.1

a Mode 9 was removed.

### Cross-Validation

In addition to testing larger molecules from outside the F1 set, the transferability of the WPS factors has also been tested using cross-validation. Here, the molecules in the modified F1 set have been randomly split into training and test sets using ratios of 9:1, 8:2, and 7:3, rounded to the nearest molecule. New power law scaling factors and frequency errors have been calculated using the reduced number of molecules in the training sets with the B97/Def2-TZVPD method, and the new scaling factors have then been used to calculate scaled frequencies and errors for the molecules in the test set. This process was repeated 100 times for each of the training to test set ratios, and the results are shown in Table 10. The average RMSDs of the training set are relatively unchanged by the different split ratios, and fall between 15.2 and 15.3 cm^–1^, with the average RMSDs of the test set ranging between 14.8 and 15.5 cm^–1^. Scaling factors are affected in the third decimal place for λ_1_, and in the fourth decimal place for λ_2_. Removing up to 30% of the training data does not impact either the scaling factors or their errors from experiment beyond these levels and indicates that the WPS method is relatively stable with respect to data set reduction, capturing a genuine trend rather than overfitting to any subset of the training data.

**Table 10:** Average Cross-Validation Scaling Factors, Average RMSDs (in cm^–1^), and Standard Deviations

	scaling factors			training set			test set		
λ_1_	σ_λ1_	λ_2_	σ_λ2_	%	RMSD	σ_RMSD_	%	RMSD	σ_RMSD_
1.177216		0.975198		100	15.3		0		
1.178485	0.002559	0.975058	0.000281	90	15.3	0.3	10	14.8	2.2
1.178053	0.003941	0.975107	0.000432	80	15.2	0.4	20	15.5	1.7
1.178640	0.004395	0.975047	0.000490	70	15.3	0.5	30	15.2	1.2

## Conclusions

Power law harmonic frequency scaling factors have been derived for the fundamental vibrational frequencies, enthalpy contributions, and entropy contributions of a range of molecules for more than 500 levels of electronic structure theory. The best-performing method here is B97/Def2-TZVPD, which gives an RMSD of 15.3 cm^–1^ and MED of 6.9 cm^–1^, corresponding to ca. 40% and ca. 64% improvements over uniform scaling, respectively. WPS frequencies significantly improve on uniform scaling in almost all cases and can describe anharmonic effects more evenly across the full spectral range. WPS frequencies converge faster with respect to basis-set size than uniform scaling, and WPS can improve GGA DFT methods that are resistant to uniform scaling. WPS also captures both fundamental vibrational frequencies and thermodynamic properties using a single unified set of scaling factors. The B97, B97–1, and the τ-HCTHh DFT methods in combination with the Def2-TZVPD basis set are found to match the experimental data most closely and are recommended for the calculation of vibrational properties.

## Supplementary Material





## Data Availability

The quantum chemical calculations were performed using a developmental version of the Q-Chem 6 software package (https://www.q-chem.com). The modified F1 experimental vibrational database, integration grid selections, and complete scaling factor data sets are provided explicitly in the . All other computational data is available from the corresponding author upon reasonable request.

## Notes

Details of the experimental vibrational frequencies, integration grids, scaling factors, and scaling factor accuracies (PDF)
Additional scaling factors (XLSX)

## References

[1] Wilson E. B. (1939). A method
of obtaining the expanded secular equation for the vibration frequencies
of a molecule. J. Chem. Phys..

[2] Pople J. A., Schlegel H. B., Krishnan R., Defrees D. J., Binkley J. S., Frisch M. J., Whiteside R. A., Hout R. F., Hehre W. J. (1981). Molecular
orbital studies of vibrational frequencies. Int. J. Quantum Chem..

[3] Defrees D. J., Mclean A. D. (1985). Molecular-Orbital
Predictions of the Vibrational Frequencies
of Some Molecular-Ions. J. Chem. Phys..

[4] Andersson M.
P., Uvdal P. (2005). New scale
factors for harmonic vibrational frequencies using the
B3LYP density functional method with the triple-zeta basis set 6–311+G­(d,p). J. Phys. Chem. A.

[5] Tantirungrotechai Y., Phanasant K., Roddecha S., Surawatanawong P., Sutthikhum V., Limtrakul J. (2006). Scaling factors for vibrational frequencies
and zero-point vibrational energies of some recently developed exchange-correlation
functionals. J. Mol. Struct.:THEOCHEM.

[6] Hanson-Heine M. W. D., George M. W., Besley N. A. (2012). Investigating
the calculation of
anharmonic vibrational frequencies using force fields derived from
density functional theory. J. Phys. Chem. A.

[7] Friese D. H., Törk L., Hättig C. (2014). Vibrational frequency scaling factors
for correlation consistent basis sets and the methods CC2 and MP2
and their spin-scaled SCS and SOS variants. J. Chem. Phys..

[8] Summers P. A., Dawson J., Ghiotto F., Hanson-Heine M. W. D., Vuong K. Q., Davies E. S., Sun X.-Z., Besley N. A., McMaster J., George M. W., Schroeder M. (2014). Photochemical
Dihydrogen Production Using an Analogue of the Active Site of NiFe
Hydrogenase. Inorg. Chem..

[9] Assefa M. K., Devera J. L., Brathwaite A. D., Mosley J. D., Duncan M. A. (2015). Vibrational
scaling factors for transition metal carbonyls. Chem. Phys. Lett..

[10] Kesharwani M. K., Brauer B., Martin J. M. L. (2015). Frequency and
Zero-Point Vibrational
Energy Scale Factors for Double-Hybrid Density Functionals (and Other
Selected Methods): Can Anharmonic Force Fields Be Avoided?. J. Phys. Chem. A.

[11] Hanson-Heine M. W. D. (2018). Uncontracted
core Pople basis sets in vibrational frequency calculations. Int. J. Quantum Chem..

[12] Hanson-Heine M. W. D. (2019). Benchmarking
DFT-D Dispersion Corrections for Anharmonic Vibrational Frequencies
and Harmonic Scaling Factors. J. Phys. Chem.
A.

[13] Hanson-Heine M. W. D. (2020). Static
correlation in vibrational frequencies studied using thermally-assisted-occupation
density functional theory. Chem. Phys. Lett..

[14] Hanson-Heine M. W. D. (2022). Static
Electron Correlation in Anharmonic Molecular Vibrations: A Hybrid
TAO-DFT Study. J. Phys. Chem. A.

[15] Tikhonov D. S., Gordiy I., Iakovlev D. A., Gorislav A. A., Kalinin M. A., Nikolenko S. A., Malaskeevich K. M., Yureva K., Matsokin N. A., Schnell M. (2024). Harmonic Scale Factors of Fundamental Transitions for
Dispersion-corrected Quantum Chemical Methods. ChemPhysChem.

[16] Sinha P., Boesch S. E., Gu C., Wheeler R. A., Wilson A. K. (2004). Harmonic
Vibrational Frequencies: Scaling Factors for HF, B3LYP, and MP2 Methods
in Combination with Correlation Consistent Basis Sets. J. Phys. Chem. A.

[17] Fekete Z. A., Hoffmann E. A., Körtvélyesi T., Penke B. (2007). Harmonic vibrational
frequency scaling factors for the new NDDO Hamiltonians: RM1 and PM6. Mol. Phys..

[18] Halls M. D., Velkovski J., Schlegel H. B. (2001). Harmonic frequency scaling factors
for Hartree-Fock, S-VWN, B-LYP, B3-LYP, B3-PW91 and MP2 with the Sadlej
pVTZ electric property basis set. Theor. Chem.
Acc..

[19] Zapata
Trujillo J. C., McKemmish L. K. (2022). Meta-analysis of uniform scaling
factors for harmonic frequency calculations. Wiley Interdiscip. Rev. Comput. Mol. Sci..

[20] Kashinski D. O., Chase G. M., Nelson R. G., Di Nallo O. E., Scales A. N., VanderLey D. L., Byrd E. F. C. (2017). Harmonic Vibrational Frequencies:
Approximate Global Scaling Factors for TPSS, M06, and M11 Functional
Families Using Several Common Basis Sets. J.
Phys. Chem. A.

[21] Alecu I. M., Zheng J., Zhao Y., Truhlar D. G. (2010). Computational Thermochemistry:
Scale Factor Databases and Scale Factors for Vibrational Frequencies
Obtained from Electronic Model Chemistries. J. Chem. Theory Comput..

[22] Zapata
Trujillo J. C., McKemmish L. K. (2023). Model Chemistry Recommendations for
Scaled Harmonic Frequency Calculations: A Benchmark Study. J. Phys. Chem. A.

[23] Witek H. A., Morokuma K. (2004). Systematic study of
vibrational frequencies calculated
with the self-consistent charge density functional tight-binding method. J. Comput. Chem..

[24] Laury M. L., Boesch S. E., Haken I., Sinha P., Wheeler R. A., Wilson A. K. (2011). Harmonic vibrational
frequencies: Scale factors for
pure, hybrid, hybrid meta, and double-hybrid functionals in conjunction
with correlation consistent basis sets. J. Comput.
Chem..

[25] Laury M. L., Carlson M. J., Wilson A. K. (2012). Vibrational frequency scale factors
for density functional theory and the polarization consistent basis
sets. J. Comput. Chem..

[26] Ünal Y., Nassif W., Özaydin B. C., Sayin K. (2021). Scale factor database
for the vibration frequencies calculated in M06–2X, one of
the DFT methods. Vib. Spectrosc..

[27] Miguel B., Brémond E., Pérez-Jiménez A. J., Adamo C., Sancho-García J. C. (2026). Accuracy and Scaling
Factors of Non-Empirical Double-Hybrid Density Functionals for Harmonic
and Fundamental Frequencies (And ZPVE). J. Comput.
Chem..

[28] Wong M. W. (1996). Vibrational
frequency prediction using density functional theory. Chem. Phys. Lett..

[29] Pople J. A., Scott A. P., Wong M. W., Radom L. (1993). Scaling Factors for
Obtaining Fundamental Vibrational Frequencies and Zero-Point Energies
from HF/6–31G* and MP2/6–31G* Harmonic Frequencies. Isr. J. Chem..

[30] Scott A. P., Radom L. (1996). Harmonic vibrational
frequencies: An evaluation of Hartree-Fock,
Møller-Plesset, quadratic configuration interaction, density
functional theory, and semiempirical scale factors. J. Phys. Chem. A.

[31] Merrick J. P., Moran D., Radom L. (2007). An evaluation
of harmonic vibrational
frequency scale factors. J. Phys. Chem. A.

[32] Hanson-Heine M. W. D., George M. W., Besley N. A. (2013). Calculating excited state properties
using Kohn-Sham density functional theory. J.
Chem. Phys..

[33] Hanson-Heine M. W. D., Wriglesworth A., Uroos M., Calladine J. A., Murphy T. S., Hamilton M., Clark I. P., Towrie M., Dowden J., Besley N. A., George M. W. (2015). Calculating singlet
excited states: Comparison with fast time-resolved infrared spectroscopy
of coumarins. J. Chem. Phys..

[34] Hanson-Heine M. W. D., George M. W., Besley N. A. (2019). Electronically excited state geometries
and vibrational frequencies calculated using the algebraic diagrammatic
construction scheme for the polarization propagator. Chem. Phys. Lett..

[35] Liu J., Liang W. (2011). Analytical approach for the excited-state Hessian in
time-dependent
density functional theory: Formalism, implementation, and performance. J. Chem. Phys..

[36] Irikura K. K., Johnson R. D., Kacker R. N. (2005). Uncertainties in Scaling Factors
for ab Initio Vibrational Frequencies. J. Phys.
Chem. A.

[37] Irikura K. K., Johnson R. D., Kacker R. N., Kessel R. (2009). Uncertainties
in scaling factors for ab initio vibrational zero-point energies. J. Chem. Phys..

[38] Pernot P., Cailliez F. (2011). Comment on “Uncertainties
in scaling factors
for ab initio vibrational zero-point energies” [J. Chem. Phys.
130, 114102 (2009)] and “Calibration sets and the accuracy
of vibrational scaling factors: A case study with the X3LYP hybrid
functional” [J. Chem. Phys. 133, 114109 (2010)]. J. Chem. Phys..

[39] Teixeira F., Melo A., Cordeiro M. N. D. S. (2011). Response
to “Comment on ‘Uncertainties
in scaling factors for ab initio vibrational zero-point energies’
and ‘Calibration sets and the accuracy of vibrational scaling
factors: A case study with the X3LYP hybrid functional’”
[J. Chem. Phys. 134, 167101 (2011)]. J. Chem.
Phys..

[40] Irikura K. K., Johnson R. D., Kacker R. N., Kessel R. (2011). Response to
“Comment on ‘Uncertainties in scaling factors for ab
initio vibrational zero-point energies’ and ‘Calibration
sets and the accuracy of vibrational scaling factors: A case study
with the X3LYP hybrid functional’” [J. Chem. Phys. 134,
167101 (2011)]. J. Chem. Phys..

[41] Wang J., Zhang C., Li Y., Zhou Y., Shu Y., Liang S., Zhang G., Liu Z., Wang Y. (2024). Performance
of Minnesota Functionals on Vibrational Frequency. Int. J. Quantum Chem..

[42] Grev R. S., Janssen C. L., Schaefer H. F. (1991). Concerning zero-point
vibrational energy corrections to electronic energies. J. Chem. Phys..

[43] Csonka G. I., Ruzsinszky A., Perdew J. P. (2005). Estimation, Computation, and Experimental
Correction of Molecular Zero-Point Vibrational Energies. J. Phys. Chem. A.

[44] Yoshida H., Ehara A., Matsuura H. (2000). Density functional
vibrational analysis
using wavenumber-linear scale factors. Chem.
Phys. Lett..

[45] Yoshida H., Takeda K., Okamura J., Ehara A., Matsuura H. (2002). A New Approach
to Vibrational Analysis of Large Molecules by Density Functional Theory:
Wavenumber-Linear Scaling Method. J. Phys. Chem.
A.

[46] Sibaev M., Crittenden D. L. (2015). Quadratic Corrections to Harmonic Vibrational Frequencies
Outperform Linear Models. J. Phys. Chem. A.

[47] Berezin K. V., Nechaev V. V., Krivokhizhina T. V. (2003). Application of a method of linear
scaling of frequencies in calculations of the normal vibrations of
polyatomic molecules. Opt. Spectrosc..

[48] Thul P., Gupta V. P., Ram V. J., Tandon P. (2010). Structural and spectroscopic
studies on 2-pyranones. Spectrochim. Acta, Part
A.

[49] Harada T., Yoshida H., Ohno K., Matsuura H. (2002). Conformational
stabilities
of 1-methoxy-2-(methylthio)­ethane and relevant intramolecular CH···O
interaction studied by matrix-isolation infrared spectroscopy and
density functional calculations. Chem. Phys.
Lett..

[50] Palafox M. A. (2018). DFT computations
on vibrational spectra: Scaling procedures to improve the wavenumbers. Phys. Sci. Rev..

[51] Hanson-Heine M. W. D. (2026). Vibrational
scaling factors follow a power law. Spectrochim.
Acta, Part A.

[52] Pulay P., Fogarasi G., Pongor G., Boggs J. E., Vargha A. (1983). Combination
of theoretical ab initio and experimental information to obtain reliable
harmonic force constants. Scaled quantum mechanical (QM) force fields
for glyoxal, acrolein, butadiene, formaldehyde, and ethylene. J. Am. Chem. Soc..

[53] Rauhut G., Pulay P. (1995). Transferable Scaling
Factors for Density Functional Derived Vibrational
Force Fields. J. Phys. Chem. A.

[54] Kalincsák F., Pongor G. (2002). Extension of the density functional derived scaled
quantum mechanical force field procedure. Spectrochim.
Acta, Part A.

[55] Baker J., Jarzecki A. A., Pulay P. (1998). Direct Scaling of Primitive
Valence
Force Constants: An Alternative Approach to Scaled Quantum Mechanical
Force Fields. J. Phys. Chem. A.

[56] Borowski P., Fernández-Gómez M., Fernández-Liencres M.-P., Ruiz T. P. (2007). An effective scaling
frequency factor method for scaling
of harmonic vibrational frequencies: theory and preliminary application
to toluene. Chem. Phys. Lett..

[57] Borowski P., Drzewiecka A., Fernández-Gómez M., Fernández-Liencres M. P., Ruiz T. P. (2008). An effective scaling
frequency factor method for harmonic vibrational frequencies: The
factors’ transferability problem. Chem.
Phys. Lett..

[58] Borowski P. (2010). An effective
scaling frequency factor method for scaling of harmonic vibrational
frequencies: The use of redundant primitive coordinates. J. Mol. Spectrosc..

[59] Borowski P., Pilorz K., Pitucha M. (2010). An effective
scaling frequency factor
method for scaling of harmonic vibrational frequencies: Application
to 1,2,4-triazole derivatives. Spectrochim.
Acta, Part A.

[60] Borowski P., Ruiz T. P., Barczak M., Pilorz K., Pasieczna-Patkowska S. (2012). Application
of the multi-parameter SQM harmonic force field, and ESFF harmonic
frequencies scaling procedures to the determination of the vibrational
spectra of silicon- and sulfur­(II)-containing compounds. Spectrochim. Acta, Part A.

[61] Irikura K. K. (2005). Mass scaling
for vibrational frequencies from ab initio calculations. Chem. Phys. Lett..

[62] Johnson R. D., Irikura K. K., Kacker R. N., Kessel R. (2010). Scaling Factors
and Uncertainties for ab Initio Anharmonic Vibrational Frequencies. J. Chem. Theory Comput..

[63] Khanifaev J., Schrader T., Perlt E. (2024). Machine-learning to predict anharmonic
frequencies: a study of models and transferability. Phys. Chem. Chem. Phys..

[64] Käser S., Boittier E. D., Upadhyay M., Meuwly M. (2021). Transfer Learning to
CCSD­(T): Accurate Anharmonic Frequencies from Machine Learning Models. J. Chem. Theory Comput..

[65] Lam J., Abdul-Al S., Allouche A.-R. (2020). Combining
Quantum Mechanics and Machine-Learning
Calculations for Anharmonic Corrections to Vibrational Frequencies. J. Chem. Theory Comput..

[66] Khanifaev J., Schrader T., Perlt E. (2024). The effect
of machine learning predicted
anharmonic frequencies on thermodynamic properties of fluid hydrogen
fluoride. J. Chem. Phys..

[67] Abdul
Al S., Allouche A.-R. (2024). Neural network approach for predicting infrared spectra
from 3D molecular structure. Chem. Phys. Lett..

[68] Miotto M., Monacelli L. (2024). Fast prediction
of anharmonic vibrational spectra for
complex organic molecules. NPJ Comput. Mater..

[69] Epifanovsky E., Gilbert A. T. B., Feng X., Lee J., Mao Y., Mardirossian N., Pokhilko P., White A. F., Coons M. P., Dempwolff A. L. (2021). Software for the frontiers
of quantum chemistry:
An overview of developments in the Q-Chem 5 package. J. Chem. Phys..

[70] Hartree D. R. (1928). The wave
mechanics of an atom with a non-Coulomb central field Part I theory
and methods. Math. Proc. Cambridge Philos. Soc..

[71] Fock V. (1930). Approximation
method for the solution of the quantum mechanical multibody problems. Z. Phys..

[72] Slater J. C. (1930). Note on
Hartree’s method. Phys. Rev..

[73] Møller C., Plesset M. S. (1934). Note on an approximation treatment
for many-electron
systems. Phys. Rev..

[74] Purvis G. D., Bartlett R. J. (1982). A full coupled-cluster singles and
doubles model: The inclusion of disconnected triples. J. Chem. Phys..

[75] Becke A. D. (1988). Density-functional
exchange-energy approximation with correct asymptotic behavior. Phys. Rev. A.

[76] Lee C. T., Yang W. T., Parr R. G. (1988). Development
of the Colle-Salvetti
correlation-energy formula into a functional of the electron density. Phys. Rev. B.

[77] Perdew J. P. (1986). Density-functional
approximation for the correlation energy of the inhomogeneous electron
gas. Phys. Rev. B.

[78] Adamson R. D., Gill P. M. W., Pople J. A. (1998). Empirical
density functionals. Chem. Phys. Lett..

[79] Hamprecht F. A., Cohen A. J., Tozer D. J., Handy N. C. (1998). Development
and
assessment of new exchange-correlation functionals. J. Chem. Phys..

[80] Boese A. D., Doltsinis N. L., Handy N. C., Sprik M. (2000). New generalized gradient
approximation functionals. J. Chem. Phys..

[81] Boese A. D., Handy N. C. (2001). A new parametrization
of exchange-correlation generalized
gradient approximation functionals. J. Chem.
Phys..

[82] Keal T. W., Tozer D. J. (2004). A semiempirical generalized gradient approximation
exchange-correlation functional. J. Chem. Phys..

[83] Handy N. C., Cohen A. J. (2001). Left-right correlation
energy. Mol. Phys..

[84] Perdew J. P., Burke K., Ernzerhof M. (1996). Generalized
Gradient Approximation
Made Simple. Phys. Rev. Lett..

[85] Mardirossian N., Head-Gordon M. (2015). Mapping the genome of meta-generalized gradient approximation
density functionals: The search for B97M-V. J. Chem. Phys..

[86] Bartók A. P., Yates J. R. (2019). Regularized SCAN functional. J. Chem. Phys..

[87] Sun J., Ruzsinszky A., Perdew J. P. (2015). Strongly Constrained and Appropriately
Normed Semilocal Density Functional. Phys. Rev.
Lett..

[88] Boese A. D., Handy N. C. (2002). New exchange-correlation
density functionals: The role
of the kinetic-energy density. J. Chem. Phys..

[89] Tao J., Perdew J. P., Staroverov V. N., Scuseria G. E. (2003). Climbing the Density
Functional Ladder: Nonempirical Meta-Generalized Gradient Approximation
Designed for Molecules and Solids. Phys. Rev.
Lett..

[90] Van
Voorhis T., Scuseria G. E. (1998). A novel form for the exchange-correlation
energy functional. J. Chem. Phys..

[91] Adamo C., Barone V. (1997). Toward reliable adiabatic
connection models free from
adjustable parameters. Chem. Phys. Lett..

[92] Becke A. D. (1993). Density-functional
thermochemistry. III. The role of exact exchange. J. Chem. Phys..

[93] Stephens P. J., Devlin F. J., Chabalowski C. F., Frisch M. J. (1994). Ab-Initio calculation
of vibrational absorption and circular-dichroism spectra using density-functional
force-fields. J. Phys. Chem. A.

[94] Schmider H. L., Becke A. D. (1998). Optimized density functionals from the extended G2
test set. J. Chem. Phys..

[95] Becke A. D. (1997). Density-functional
thermochemistry. V. Systematic optimization of exchange-correlation
functionals. J. Chem. Phys..

[96] Boese A. D., Martin J. M. L. (2004). Development of
density functionals for thermochemical
kinetics. J. Chem. Phys..

[97] Wilson P. J., Bradley T. J., Tozer D. J. (2001). Hybrid
exchange-correlation functional
determined from thermochemical data and ab initio potentials. J. Chem. Phys..

[98] Keal T. W., Tozer D. J. (2005). Semiempirical hybrid
functional with improved performance
in an extensive chemical assessment. J. Chem.
Phys..

[99] Becke A. D. (1993). A new mixing
of Hartree–Fock and local density-functional theories. J. Chem. Phys..

[100] Lin C. Y., George M. W., Gill P. M. W. (2004). EDF2:
A density
functional for predicting molecular vibrational frequencies. Aust. J. Chem..

[101] Lynch B. J., Fast P. L., Harris M., Truhlar D. G. (2000). Adiabatic
Connection for Kinetics. J. Phys. Chem. A.

[102] Adamo C., Barone V. (1998). Exchange functionals
with improved
long-range behavior and adiabatic connection methods without adjustable
parameters: The mPW and mPW1PW models. J. Chem.
Phys..

[103] Hoe W.-M., Cohen A. J., Handy N. C. (2001). Assessment of a
new local exchange functional OPTX. Chem. Phys.
Lett..

[104] Adamo C., Barone V. (1999). Toward reliable density functional
methods without adjustable parameters: The PBE0 model. J. Chem. Phys..

[105] Xu X., Goddard W. A. (2004). The X3LYP extended
density functional for accurate
descriptions of nonbond interactions, spin states, and thermochemical
properties. Proc. Natl. Acad. Sci. U.S.A..

[106] Zhao Y., Schultz N. E., Truhlar D. G. (2005). Exchange-correlation
functional with broad accuracy for metallic and nonmetallic compounds,
kinetics, and noncovalent interactions. J. Chem.
Phys..

[107] Zhao Y., Schultz N. E., Truhlar D. G. (2006). Design
of Density
Functionals by Combining the Method of Constraint Satisfaction with
Parametrization for Thermochemistry, Thermochemical Kinetics, and
Noncovalent Interactions. J. Chem. Theory Comput..

[108] Zhao Y., Truhlar D. G. (2008). The M06 suite of
density functionals
for main group thermochemistry, thermochemical kinetics, noncovalent
interactions, excited states, and transition elements: two new functionals
and systematic testing of four M06-class functionals and 12 other
functionals. Theor. Chem. Acc..

[109] Zhao Y., Truhlar D. G. (2008). Exploring the Limit of Accuracy of
the Global Hybrid Meta Density Functional for Main-Group Thermochemistry,
Kinetics, and Noncovalent Interactions. J. Chem.
Theory Comput..

[110] Quintal M. M., Karton A., Iron M. A., Boese A. D., Martin J. M. L. (2006). Benchmark
Study of DFT Functionals for Late-Transition-Metal
Reactions. J. Phys. Chem. A.

[111] Yanai T., Tew D. P., Handy N. C. (2004). A new hybrid
exchange-correlation
functional using the Coulomb-attenuating method (CAM-B3LYP). Chem. Phys. Lett..

[112] Krukau A. V., Vydrov O. A., Izmaylov A. F., Scuseria G. E. (2006). Influence
of the exchange screening parameter on the performance of screened
hybrid functionals. J. Chem. Phys..

[113] Henderson T. M., Janesko B. G., Scuseria G. E. (2008). Generalized gradient
approximation model exchange holes for range-separated hybrids. J. Chem. Phys..

[114] Mardirossian N., Ruiz Pestana L., Womack J. C., Skylaris C.-K., Head-Gordon T., Head-Gordon M. (2017). Use of the rVV10 Nonlocal Correlation
Functional in the B97M-V Density Functional: Defining B97M-rV and
Related Functionals. J. Phys. Chem. Lett..

[115] Chai J.-D., Head-Gordon M. (2008). Long-range corrected hybrid density
functionals with damped atom–atom dispersion corrections. Phys. Chem. Chem. Phys..

[116] Lin Y.-S., Li G.-D., Mao S.-P., Chai J.-D. (2013). Long-Range
Corrected Hybrid Density Functionals with Improved Dispersion Corrections. J. Chem. Theory Comput..

[117] Mardirossian N., Head-Gordon M. (2014). ωB97X-V:
A 10-parameter, range-separated
hybrid, generalized gradient approximation density functional with
nonlocal correlation, designed by a survival-of-the-fittest strategy. Phys. Chem. Chem. Phys..

[118] Hehre W. J., Ditchfie R., Pople J. A. (1972). Self-Consistent
Molecular Orbital Methods. XII. Further Extensions of Gaussian-Type
Basis Sets for Use in Molecular Orbital Studies of Organic Molecules. J. Chem. Phys..

[119] Hariharan P. C., Pople J. A. (1973). The influence of polarization functions
on molecular orbital hydrogenation energies. Theor. Chim. Acta.

[120] Clark T., Chandrasekhar J., Spitznagel G. W., Schleyer P. V. R. (1983). Efficient diffuse
function-augmented basis sets for
anion calculations. III. The 3–21+G basis set for first-row
elements, Li–F. J. Comput. Chem..

[121] Krishnan R., Binkley J. S., Seeger R., Pople J. A. (1980). Self-consistent
molecular orbital methods. XX. A basis set for correlated wave functions. J. Chem. Phys..

[122] Frisch M. J., Pople J. A., Binkley J. S. (1984). Self-Consistent
molecular orbital methods 25. Supplementary functions for gaussian
basis sets. J. Chem. Phys..

[123] Dunning T. H. (1989). Gaussian basis
sets for use in correlated
molecular calculations. I. The atoms boron through neon and hydrogen. J. Chem. Phys..

[124] Kendall R. A., Dunning T. H., Harrison R. J. (1992). Electron
affinities of the first-row atoms revisited. Systematic basis sets
and wave functions. J. Chem. Phys..

[125] Jensen F. (2001). Polarization consistent basis sets:
Principles. J. Chem. Phys..

[126] Jensen F. (2002). Polarization consistent basis sets.
III. The importance
of diffuse functions. J. Chem. Phys..

[127] Weigend F., Ahlrichs R. (2005). Balanced basis sets
of split valence,
triple zeta valence and quadruple zeta valence quality for H to Rn:
Design and assessment of accuracy. Phys. Chem.
Chem. Phys..

[128] Rappoport D., Furche F. (2010). Property-optimized Gaussian basis
sets for molecular response calculations. J.
Chem. Phys..

[129] Zapata Trujillo J. C., McKemmish L. K. (2022). VIBFREQ1295:
A New Database for Vibrational
Frequency Calculations. J. Phys. Chem. A.

[130] Krishnadas A., Kansal J., Charron N. E., Ragyanszki A. (2026). Spectral Quantum
Chemistry and Infrared Resonance Library for Data-Driven Molecular
Spectroscopy. Sci. Data.

[131] Chien S. H., Gill P. M. W. (2006). SG-0: A small standard grid for DFT
quadrature on large systems. J. Comput. Chem..

[132] Gill P. M.
W., Johnson B. G., Pople J. A. (1993). A standard grid
for density functional calculations. Chem. Phys.
Lett..

[133] Dasgupta S., Herbert J. M. (2017). Standard grids for high-precision
integration of modern density functionals: SG-2 and SG-3. J. Comput. Chem..

[134] Microsoft Corporation . Microsoft Excel, Microsoft 365; Microsoft Corporation: Redmond, WA, 2025.

[135] Boese A. D., Klopper W., Martin J. M. L. (2005). Assessment of
various density functionals and basis sets for the calculation of
molecular anharmonic force fields. Int. J. Quantum
Chem..

[136] Biczysko M., Panek P., Scalmani G., Bloino J., Barone V. (2010). Harmonic and
Anharmonic Vibrational Frequency Calculations
with the Double-Hybrid B2PLYP Method: Analytic Second Derivatives
and Benchmark Studies. J. Chem. Theory Comput..

[137] Green J.
H. S., Harrison D. J. (1976). Vibrational
spectra of benzene derivatives—XVII.
Benzonitrile and substituted benzonitriles. Spectrochim. Acta, Part A.

[138] Martin J. M. L., El-Yazal J., François J.-P. (1996). Structure
and Vibrational Spectrum of Some Polycyclic Aromatic Compounds Studied
by Density Functional Theory. 1. Naphthalene, Azulene, Phenanthrene,
and Anthracene. J. Phys. Chem. A.

