# Supplementary material for: Evaluating Power-Law Scaling Factors for Harmonic Vibrational Frequencies across Model Chemistries
Source: J Chem Theory Comput. 2026 Aug 15;22(17):9039–53. doi: 10.1021/acs.jctc.6c01138 (PMC13564362; doi:10.1021/acs.jctc.6c01138)
Supplement: Supplementary file 1 [file ct6c01138_si_001.pdf]

Evaluating Power Law Scaling Factors for Harmonic Vibrational Frequencies Across Model Chemistries: Supporting Information

Magnus W. D. Hanson-Heine* and Adam M. Steer

School of Chemistry and Chemical Engineering, University of Southampton, Highfield, Southampton SO17 1BE, U.K.

*Email: magnus.hansonheine@soton.ac.uk

Table S1. Uniform scaling factors for fundamental vibrations.

Method	6-31G(d)	6-31+G(d,p)	6-31+G(2df,p)	6-311+G(d,p)	6-311+G(2df,p)
	λ	λ	λ	λ	λ
HF	0.896817	0.902220	0.907042	0.907408	0.908886
MP2[a]	0.944719	0.942113	0.950061	0.952703	0.955698
CCSD[b]	0.951116	0.943149	0.949234	0.953957	0.955447
BLYP	0.993367	0.996166	0.998730	0.999044	0.998626
BP86	0.990876	0.993365	0.996267	0.996971	0.968136
EDF1	0.979434	0.981744	0.984825	0.985308	0.985829
HCTH-93	0.974771	0.977087	0.980408	0.980734	0.981760
HCTH-120	0.975941	0.978601	0.981842	0.981790	0.982684
HCTH-147	0.975019	0.977481	0.980780	0.980827	0.981756
HCTH-407	0.969542	0.972409	0.976191	0.975691	0.977389
KT3	0.960742	0.962594	0.965688	0.966096	0.967414
OLYP	0.977676	0.980308	0.983939	0.983873	0.985204
PBE	0.987424	0.990279	0.993386	0.993904	0.994485
B97M-V	0.954602	0.954765	0.957693	0.958512	0.959753
rSCAN	0.961237	0.963399	0.966488	0.967539	0.968189
SCAN	0.959385	0.961527	0.963870	0.965637	0.965190
τ-HCTH	0.975080	0.976885	0.980025	0.981269	0.981976
TPSS	0.973610	0.976038	0.979946	0.981159	0.981678
VSXC	0.966562	0.968281	0.971197	0.972361	0.973504
B1LYP	0.956320	0.959989	0.963239	0.963935	0.964074
B3LYP	0.961352	0.964814	0.967967	0.968665	0.968747
B3P86	0.960246	0.963472	0.966860	0.967645	0.968136
B3PW91	0.957623	0.960637	0.964147	0.964983	0.965642
B97	0.962526	0.965769	0.969087	0.969863	0.970330
B97-K	0.943874	0.948144	0.951795	0.952603	0.953329
B97-1	0.961256	0.964628	0.967895	0.968667	0.969045
B97-2	0.951462	0.954423	0.958138	0.958888	0.959967
B97-3	0.949715	0.953214	0.956711	0.957530	0.958231
BHHLYP	0.925194	0.929508	0.933320	0.934108	0.934742
EDF2	0.959413	0.962871	0.966016	0.966803	0.966938
MPW1K	0.929022	0.932724	0.936820	0.937530	0.938775
MPW1PW91	0.950631	0.953924	0.957638	0.958465	0.959378
O3LYP	0.962189	0.965271	0.969022	0.969125	0.970436
PBE0	0.952083	0.955583	0.959356	0.959932	0.961004
X3LYP	0.959348	0.963019	0.966226	0.966919	0.967059
M05	0.949905	0.954828	0.957563	0.960777	0.962007
M05-2X	0.938531	0.943029	0.946120	0.946092	0.946431
M06	0.960153	0.963268	0.965315	0.968194	0.967630
M06-2X	0.945455	0.949670	0.952805	0.952318	0.953192
M08-HX	0.953351	0.956406	0.958391	0.959467	0.960098
M08-SO	0.955390	0.961035	0.963738	0.967278	0.967644
τ-HCTHh	0.966437	0.969280	0.972400	0.973294	0.973441
TPSS0	0.943148	0.946388	0.950616	0.951560	0.952557
CAM-B3LYP	0.949066	0.953218	0.956711	0.957301	0.957668
HSE-HJS	0.952621	0.956125	0.959858	0.960442	0.961473
LC-rVV10	0.930745	0.934890	0.939311	0.939405	0.940963
ωB97X-D	0.949959	0.953234	0.957214	0.957761	0.958744
ωB97X-D3	0.947131	0.950687	0.954729	0.955262	0.956410
ωB97X-V	0.947141	0.950979	0.955041	0.955357	0.956603

[a]MP2 problem cases removed. [b]ClCCCl and HCCCCH removed.

Table S2. Uniform scaling factors for fundamental vibrations (cont.)

Method	6-31++G(d,p)	6-311++G(d,p)	6-311++G(2df,p)	cc-pVTZ	aug-cc-pVTZ
	λ	λ	λ	λ	λ
HF	0.902319	0.907454	0.908940	0.908760	0.909174
MP2[a]	0.942661	0.953121	0.955843	0.953917	0.956910
CCSD[b]	0.943663	0.953132	0.955577	0.953417	-
BLYP	0.996359	0.999140	0.998699	0.998313	0.998926
BP86	0.993561	0.997063	0.997168	0.997038	0.997730
EDF1	0.981998	0.985426	0.985923	0.986092	0.986733
HCTH-93	0.977364	0.980851	0.981838	0.982243	0.982782
HCTH-120	0.978817	0.981860	0.982736	0.982957	0.983482
HCTH-147	0.977710	0.980918	0.981814	0.982080	0.982586
HCTH-407	0.972613	0.975764	0.977419	0.977970	0.978566
KT3	0.962864	0.966189	0.967441	0.965816	0.967001
OLYP	0.980644	0.984006	0.985288	0.986273	0.987114
PBE	0.990512	0.993994	0.994559	0.994934	0.995696
B97M-V	0.954955	0.958570	0.959803	0.957565	0.957742
rSCAN	0.963577	0.967625	0.968252	0.967682	0.968194
SCAN	0.961612	0.965700	0.965156	0.966172	0.966135
τ-HCTH	0.977112	0.981359	0.982037	0.980821	0.981171
TPSS	0.976276	0.981265	0.981762	0.981900	0.982325
VSXC	0.968509	0.972448	0.973560	0.972238	0.972878
B1LYP	0.960149	0.964000	0.964131	0.963663	0.964187
B3LYP	0.964973	0.968733	0.968802	0.968410	0.968943
B3P86	0.963621	0.967714	0.968201	0.968006	0.968598
B3PW91	0.960814	0.965063	0.968201	0.965694	0.966307
B97	0.965952	0.969939	0.970392	0.970799	0.971382
B97-K	0.948301	0.952663	0.953384	0.953814	0.954381
B97-1	0.964806	0.968737	0.969101	0.969498	0.970074
B97-2	0.954613	0.958968	0.960031	0.959809	0.960306
B97-3	0.953354	0.957588	0.958287	0.958217	0.958713
BHHLYP	0.929645	0.934156	0.934791	0.934326	0.934781
EDF2	0.963031	0.966873	0.966991	0.966637	0.967197
MPW1K	0.932875	0.937608	0.938839	0.938746	0.939329
MPW1PW91	0.954108	0.958555	0.959450	0.959359	0.960011
O3LYP	0.965544	0.969236	0.970506	0.971289	0.971981
PBE0	0.955764	0.960012	0.961069	0.961282	0.961912
X3LYP	0.963178	0.966987	0.967114	0.966705	0.967260
M05	0.955042	0.960846	0.962052	0.960230	0.961240
M05-2X	0.943156	0.946132	0.946478	0.945303	0.946018
M06	0.963396	0.968268	0.967733	0.966656	0.967910
M06-2X	0.949807	0.952380	0.953246	0.952681	0.953336
M08-HX	0.956580	0.959590	0.960203	0.957574	0.958754
M08-SO	0.961172	0.967344	0.967717	0.965534	0.966993
τ-HCTHh	0.969463	0.973372	0.973508	0.973468	0.973991
TPSS0	0.946572	0.951638	0.952632	0.952668	0.953117
CAM-B3LYP	0.953366	0.957359	0.957721	0.957271	0.957882
HSE-HJS	0.956300	0.960517	0.961535	0.961734	0.962363
LC-rVV10	0.935032	0.939472	0.941035	0.941338	0.942171
ωB97X-D	0.953365	0.957812	0.958801	0.957136	0.957820
ωB97X-D3	0.950830	0.955316	0.956469	0.954947	0.955656
ωB97X-V	0.951140	0.955417	0.956661	0.956397	0.957021

[a]MP2 problem cases removed. [b]ClCCCl and HCCCCH removed.

Table S3. Uniform scaling errors for scaled fundamental vibrations (in cm^{-1}).

Method	6-31G(d)			6-31+G(d,p)			6-31+G(2df,p)			6-311+G(d,p)			6-311+G(2df,p)		
	RMSD	MAD	MED	RMSD	MAD	MED	RMSD	MAD	MED	RMSD	MAD	MED	RMSD	MAD	MED
HF	41.2	29.1	21.2	42.4	28.7	19.1	44.6	30.3	19.6	45.0	30.1	19.5	45.9	31.8	21.3
MP2[a]	40.5	27.3	18.4	49.1	36.1	27.8	42.4	32.7	26.5	43.1	30.9	22.5	36.5	26.4	19.9
CCSD[b]	24.7	18.5	15.0	29.4	23.1	19.5	23.5	19.3	17.5	24.6	18.4	14.4	19.5	14.6	11.7
BLYP	38.2	29.7	23.5	42.9	33.9	28.2	40.0	32.4	27.6	43.6	32.9	24.9	37.6	29.0	23.5
BP86	32.9	26.0	21.3	38.2	32.5	29.9	37.2	32.2	30.6	38.7	32.0	28.3	26.5	22.2	20.8
EDF1	33.4	26.3	22.0	39.2	33.3	30.1	38.0	32.8	30.4	39.4	32.4	28.3	34.5	29.2	26.4
HCTH-93	31.8	25.2	21.0	38.4	32.6	29.7	38.0	32.4	30.0	38.7	31.9	28.0	34.5	29.0	26.0
HCTH-120	32.2	25.6	21.7	38.5	32.8	30.6	37.8	32.5	30.7	38.9	32.3	28.6	34.4	29.2	26.8
HCTH-147	32.3	25.7	21.6	38.6	32.9	30.7	38.0	32.7	31.0	38.9	32.4	28.6	34.4	29.3	27.2
HCTH-407	33.8	27.0	22.8	40.5	34.4	32.9	40.3	34.5	33.0	40.7	34.1	31.5	36.9	31.3	29.3
KT3	35.1	28.9	26.2	40.9	36.1	34.2	39.4	35.0	34.0	40.8	35.6	33.3	36.9	32.7	30.9
OLYP	33.6	26.6	22.5	39.9	34.2	31.5	39.1	33.8	31.4	40.2	33.7	29.9	36.1	30.7	27.4
PBE	33.6	26.8	22.8	39.3	33.8	31.6	38.9	33.7	32.9	39.6	33.3	31.0	35.2	30.3	29.1
B97M-V	30.9	22.9	17.0	35.2	28.7	24.7	33.7	27.1	23.3	33.6	26.8	22.3	29.5	23.3	19.7
rSCAN	26.6	21.0	18.1	32.6	27.7	26.1	32.2	27.6	26.9	31.7	26.1	23.4	27.3	22.8	21.2
SCAN	33.2	23.9	17.5	38.6	29.9	24.5	36.0	28.7	25.6	37.6	28.4	23.1	34.2	25.8	20.4
τ-HCTH	30.6	24.0	20.4	36.0	30.5	28.3	34.7	30.0	28.4	35.8	29.6	26.2	31.3	26.5	23.8
TPSS	35.6	28.0	23.6	37.8	30.3	26.5	34.4	28.1	25.0	36.3	27.7	22.3	31.4	24.6	20.9
VSXC	36.1	29.0	25.7	44.4	37.9	35.4	43.1	37.2	35.0	44.2	36.8	32.5	39.4	33.5	30.8
B1LYP	25.4	19.5	16.5	28.3	23.3	20.1	27.0	22.8	20.2	28.2	22.2	17.8	23.8	19.1	15.9
B3LYP	26.2	20.3	17.3	29.7	24.9	21.5	28.3	24.3	21.9	29.7	23.8	19.6	25.0	20.5	17.6
B3P86	25.3	20.3	17.2	29.5	25.4	24.0	30.1	25.7	24.7	29.5	24.7	22.8	26.5	22.2	20.8
B3PW91	25.5	20.4	17.0	30.1	25.8	24.2	30.7	26.1	25.0	30.1	25.1	23.1	27.1	22.6	20.9
B97	24.1	19.1	16.1	28.8	24.4	22.2	28.3	24.2	22.6	29.1	23.7	20.6	25.3	21.0	19.2
B97-K	23.4	17.5	13.9	26.0	20.6	17.4	25.7	20.7	18.8	26.4	20.1	16.4	23.6	18.0	14.8
B97-1	23.9	18.9	15.8	28.4	24.0	21.8	28.0	23.9	22.6	28.7	23.3	20.4	25.0	20.6	19.0
B97-2	25.3	20.3	17.3	30.3	25.9	24.3	30.5	26.0	25.9	30.2	24.9	22.7	27.2	22.5	21.0
B97-3	23.2	18.1	15.1	27.6	22.9	20.6	28.0	23.3	21.6	27.7	22.1	18.8	24.6	19.8	17.9
BHHLYP	25.9	18.4	13.4	27.3	20.8	17.3	28.0	21.4	18.0	27.7	20.5	16.4	26.2	19.1	14.7
EDF2	26.2	20.7	17.6	30.1	25.9	23.9	29.7	25.7	24.6	29.9	24.8	21.9	25.8	21.7	20.0
MPW1K	27.9	20.7	15.9	31.2	24.7	21.1	33.0	25.9	22.3	31.8	24.3	20.4	31.2	23.8	19.7
MPW1PW91	25.5	20.3	17.1	29.9	25.3	23.7	30.9	25.9	24.9	29.9	24.7	22.2	27.7	22.6	20.7
O3LYP	28.3	22.5	19.2	33.8	29.0	26.7	33.8	28.8	27.7	34.0	28.4	25.7	30.7	25.6	23.5
PBE0	26.7	21.3	17.9	30.9	25.9	24.2	32.3	26.9	25.5	31.1	25.6	22.9	29.2	23.8	21.4
X3LYP	25.8	19.9	16.7	29.0	24.3	21.4	27.8	23.9	21.9	28.9	23.2	19.4	24.4	20.0	17.2
M05	31.4	25.7	23.4	35.6	29.8	29.2	36.7	30.5	29.2	34.7	28.0	24.9	32.9	26.4	23.8
M05-2X	31.5	24.7	19.9	32.7	27.2	25.0	33.2	28.0	26.6	30.7	25.0	22.2	29.2	23.9	22.0
M06	30.8	25.2	22.3	34.8	28.7	25.6	36.1	29.1	23.7	34.0	26.8	23.0	31.4	24.8	20.9
M06-2X	29.1	22.6	19.3	30.3	24.9	23.1	31.5	25.9	22.8	29.3	23.6	20.6	28.6	22.9	20.2
M08-HX	32.8	24.9	19.5	36.5	29.4	25.6	38.1	30.6	26.4	36.0	28.2	23.7	35.3	27.4	22.2
M08-SO	30.5	23.6	20.3	32.7	26.4	24.0	33.8	26.9	22.4	32.4	25.0	20.9	31.4	23.9	19.9
τ-HCTHh	24.8	19.8	16.8	29.6	25.3	23.6	29.0	24.9	23.7	29.7	24.5	21.6	25.6	21.6	20.3
TPSS0	24.1	18.5	15.0	25.4	20.6	18.4	24.6	20.6	18.9	24.6	19.5	16.6	21.9	17.7	15.6
CAM-B3LYP	25.3	19.1	15.2	27.2	22.5	20.4	27.2	22.8	21.8	26.7	21.4	19.0	23.9	19.2	17.0
HSE-HJS	26.6	21.2	18.0	30.8	25.9	23.8	32.1	26.8	25.5	31.0	25.6	23.0	29.0	23.7	21.3
LC-rVV10	33.5	25.6	22.7	36.4	29.4	27.8	37.9	30.9	27.8	36.1	28.9	26.1	35.6	28.5	25.6
ωB97X-D	23.4	18.0	14.3	27.7	22.5	19.6	28.3	22.9	20.8	28.1	22.0	18.5	25.7	20.2	17.7
ωB97X-D3	24.6	19.0	15.4	28.7	23.3	21.3	29.4	24.2	22.3	28.7	22.8	19.7	26.7	21.4	19.1
ωB97X-V	25.8	19.9	16.0	28.4	23.5	22.1	28.7	24.1	22.9	27.9	22.5	20.7	26.0	21.1	19.2

[a]MP2 problem cases removed. [b]ClCCCl and HCCCCH removed.

Table S4. Uniform scaling errors for scaled fundamental vibrations (in cm^{-1}) (cont.)

Method	6-31++G(d,p)			6-311++G(d,p)			6-311++G(2df,p)			cc-pVTZ			aug-cc-pVTZ		
	RMSD	MAD	MED	RMSD	MAD	MED	RMSD	MAD	MED	RMSD	MAD	MED	RMSD	MAD	MED
HF	42.4	28.7	18.9	45.0	30.1	19.5	45.9	31.8	21.4	46.3	32.2	21.0	44.9	31.3	20.7
MP2[a]	50.0	37.1	29.4	44.5	32.1	23.4	36.5	26.4	20.5	37.8	27.6	21.9	40.0	29.7	22.7
CCSD[b]	30.6	23.9	20.0	49.0	21.6	15.0	19.6	14.6	11.7	21.6	16.7	14.2	-	-	-
BLYP	42.9	33.9	28.1	43.6	33.0	25.4	37.6	29.1	23.4	36.7	28.7	23.4	39.5	30.6	25.0
BP86	38.2	32.5	30.1	38.7	32.1	28.4	33.9	28.9	26.8	33.4	28.5	26.3	35.2	30.0	27.3
EDF1	39.2	33.3	30.2	39.4	32.5	28.7	34.6	29.3	26.6	33.6	28.4	25.7	35.7	30.2	27.0
HCTH-93	38.3	32.5	29.4	38.8	32.0	28.2	34.6	29.1	26.2	33.6	28.3	25.5	35.2	29.7	26.3
HCTH-120	38.5	32.8	30.5	39.0	32.4	28.8	34.4	29.2	27.1	33.5	28.4	26.0	35.3	30.0	27.5
HCTH-147	38.6	32.9	30.7	39.0	32.5	28.8	34.5	29.4	27.3	33.7	28.6	26.5	35.4	30.1	27.5
HCTH-407	40.4	34.4	32.7	40.8	34.2	31.6	36.9	31.4	29.3	35.9	30.5	29.0	37.5	31.9	29.6
KT3	40.9	36.0	34.2	40.9	35.7	33.3	37.0	32.8	31.0	35.3	31.0	28.7	37.3	33.0	30.9
OLYP	39.8	34.1	31.4	40.3	33.8	30.3	36.1	30.8	27.9	34.3	29.1	26.3	36.3	31.0	27.8
PBE	39.3	33.8	31.6	39.6	33.4	31.1	35.3	30.4	29.1	34.4	29.5	27.7	36.3	31.2	29.2
B97M-V	35.3	28.8	24.7	33.7	26.9	22.4	29.6	23.3	19.7	30.5	24.0	19.8	31.2	25.0	21.0
rSCAN	32.6	27.7	26.1	31.8	26.2	23.5	27.4	22.9	21.2	27.2	22.9	21.8	28.4	23.9	22.2
SCAN	38.9	29.9	24.3	38.0	28.5	22.9	34.0	25.6	20.6	34.6	25.3	19.6	35.1	25.9	20.2
τ-HCTH	36.0	30.5	28.2	35.9	29.7	26.2	31.3	26.6	24.0	32.0	27.4	25.3	33.4	28.6	26.0
TPSS	37.7	30.2	26.4	36.3	27.8	22.3	31.4	24.7	20.9	30.6	24.3	21.3	32.7	25.8	22.0
VSXC	44.5	38.0	35.2	44.3	36.9	32.8	39.5	33.5	30.9	38.4	32.9	30.8	40.0	34.3	31.7
B1LYP	28.3	23.3	20.2	28.3	22.3	17.9	23.9	19.1	16.0	23.8	19.1	15.7	25.0	20.2	16.4
B3LYP	29.7	24.9	21.5	29.7	23.8	19.7	25.0	20.5	17.7	24.9	20.4	17.6	26.3	21.7	18.2
B3P86	29.6	25.4	24.1	29.6	24.8	23.1	26.5	22.2	21.0	26.6	22.3	21.4	27.1	23.0	21.6
B3PW91	30.1	25.8	24.2	30.2	25.2	23.2	26.5	22.2	21.0	27.2	22.7	21.3	27.8	23.4	21.7
B97	28.8	24.4	22.3	29.2	23.8	20.8	25.4	21.0	19.3	24.9	20.7	19.2	25.8	21.6	19.8
B97-K	26.0	20.6	17.5	26.4	20.2	16.3	23.7	18.0	14.7	23.6	17.9	14.1	23.8	18.4	14.8
B97-1	28.4	24.0	22.0	28.8	23.4	20.6	25.0	20.7	19.1	24.6	20.4	18.8	25.5	21.3	19.5
B97-2	30.3	25.9	24.4	30.2	25.0	22.7	27.3	22.6	21.0	27.3	22.8	21.7	27.9	23.5	21.7
B97-3	27.6	23.0	20.6	27.8	22.1	19.0	24.7	19.8	17.8	24.4	19.7	18.0	24.9	20.4	18.3
BHHLYP	27.3	20.8	17.4	27.7	20.5	16.3	26.3	19.2	14.7	26.6	19.4	14.2	26.0	19.2	14.6
EDF2	30.1	25.9	24.1	30.0	24.9	21.9	25.8	21.7	19.9	25.9	21.8	19.7	26.9	22.8	20.4
MPW1K	31.2	24.7	21.1	31.8	24.3	20.5	31.2	23.8	20.0	31.4	23.9	20.3	30.8	23.6	19.9
MPW1PW91	29.9	25.3	23.6	30.0	24.7	22.3	27.7	22.6	20.8	27.7	22.7	21.2	28.0	23.1	21.6
O3LYP	33.7	29.0	26.9	34.1	28.5	25.8	30.8	25.7	23.5	29.8	24.8	23.1	31.0	26.1	24.0
PBE0	30.9	26.0	24.3	31.2	25.7	23.1	29.3	23.9	21.4	29.3	23.8	21.7	29.4	24.1	22.1
X3LYP	29.0	24.4	21.3	28.9	23.3	19.4	24.5	20.1	17.3	24.3	20.0	17.3	25.7	21.2	17.9
M05	35.6	29.9	29.2	34.8	28.1	25.3	32.9	26.4	23.9	32.9	27.0	25.3	32.7	26.8	24.9
M05-2X	32.8	27.3	25.1	30.7	25.0	22.2	29.3	24.0	22.2	29.8	24.5	22.2	30.0	24.8	22.7
M06	34.9	28.8	25.6	34.1	26.9	23.1	31.5	24.8	20.8	31.4	24.6	20.4	31.0	24.8	21.0
M06-2X	30.4	24.9	23.2	29.4	23.6	20.5	28.6	22.9	20.1	28.0	22.5	20.0	27.9	22.7	20.6
M08-HX	36.5	29.4	25.5	36.1	28.3	24.0	35.4	27.5	22.4	35.2	28.3	23.9	35.3	28.4	24.2
M08-SO	32.7	26.5	23.9	32.5	25.0	21.0	31.4	24.0	20.0	30.5	23.7	19.9	30.4	23.7	20.2
τ-HCTHh	29.6	25.3	23.8	29.8	24.6	21.6	25.7	21.7	20.4	25.6	21.8	21.0	26.6	22.6	21.2
TPSS0	25.4	20.6	18.4	24.6	19.6	16.6	22.0	17.7	15.7	22.1	17.8	15.9	22.6	18.3	16.1
CAM-B3LYP	27.2	22.5	20.5	26.7	21.4	18.8	23.9	19.2	16.9	24.0	19.3	17.5	24.5	19.8	17.8
HSE-HJS	30.8	26.0	24.0	31.1	25.7	23.0	29.1	23.8	21.3	29.1	23.7	21.7	29.2	24.0	22.4
LC-rVV10	36.4	29.5	27.8	36.1	28.9	26.1	35.6	28.5	25.6	35.6	28.4	25.4	35.2	28.3	25.4
ωB97X-D	27.7	22.6	19.7	28.1	22.0	18.5	25.7	20.2	17.6	25.5	20.5	18.0	25.7	20.7	18.2
ωB97X-D3	28.7	23.4	21.5	28.7	22.8	19.6	26.8	21.5	19.3	26.4	21.6	19.6	26.7	21.9	20.0
ωB97X-V	28.4	23.6	22.2	27.9	22.5	20.8	26.0	21.1	19.2	25.8	21.0	19.2	26.1	21.5	19.4

[a]MP2 problem cases removed. [b]ClCCCl and HCCCCH removed.

Table S5. Uniform scaling factors for fundamental vibrations with additional basis sets.

Method	B3LYP	B97	B97-1	τ-HCTHh
	λ	λ	λ	λ
6-31G(d)	0.961352	0.962526	0.961256	0.966437
6-31+G(d,p)	0.964814	0.965769	0.964628	0.969280
6-31++G(d,p)	0.964973	0.965952	0.964806	0.969463
6-31+G(2df,p)	0.967967	0.969087	0.967895	0.972400
6-311+G(d,p)	0.968665	0.969863	0.968667	0.973294
6-311++G(d,p)	0.968733	0.969939	0.968737	0.973372
6-311+G(2df,p)	0.968747	0.970330	0.969045	0.973441
6-311++G(2df,p)	0.968802	0.970392	0.969101	0.973508
6-311++G(3df,3pd)	0.967458	0.969499	0.968209	0.972438
pc-2	0.967374	0.969393	0.968015	0.971882
pc-3	0.968295	0.970406	0.969037	0.973009
aug-pc-2	0.967782	0.969813	0.968464	0.972315
aug-pc-3	0.968313	0.970427	0.969055	0.973034
Def2-TZVP	0.968018	0.970011	0.968648	0.972666
Def2-TZVPD	0.968299	0.970354	0.968981	0.973034
cc-pVDZ	0.971623	0.972281	0.971159	0.976108
cc-pVTZ	0.968410	0.970799	0.969498	0.973468
cc-pVQZ	0.968540	0.970600	0.969205	0.973370
aug-cc-pVDZ	0.970665	0.972117	0.970931	0.975787
aug-cc-pVTZ	0.968943	0.971382	0.970074	0.973991
aug-cc-pVQZ	0.968618	0.970715	0.969326	0.973471

Table S6. Uniform scaling errors for fundamental vibrations (cm^{-1}) with additional basis sets.

Method	B3LYP			B97			B97-1			τ-HCTHh		
	RMSD	MAD	MED	RMSD	MAD	MED	RMSD	MAD	MED	RMSD	MAD	MED
6-31G(d)	26.2	20.3	17.3	24.1	19.1	16.1	23.9	18.9	15.8	24.8	19.8	16.8
6-31+G(d,p)	29.7	24.9	21.5	28.8	24.4	22.2	28.4	24.0	21.8	29.6	25.3	23.6
6-31++G(d,p)	29.7	24.9	21.5	28.8	24.4	22.3	28.4	24.0	22.0	29.6	25.3	23.8
6-31+G(2df,p)	28.3	24.3	21.9	28.3	24.2	22.6	28.0	23.9	22.6	29.0	24.9	23.7
6-311+G(d,p)	29.7	23.8	19.6	29.1	23.7	20.6	28.7	23.3	20.4	29.7	24.5	21.6
6-311++G(d,p)	29.7	23.8	19.7	29.2	23.8	20.8	28.8	23.4	20.6	29.8	24.6	21.6
6-311+G(2df,p)	25.0	20.5	17.6	25.3	21.0	19.2	25.0	20.6	19.0	25.6	21.6	20.3
6-311++G(2df,p)	25.0	20.5	17.7	25.4	21.0	19.3	25.0	20.7	19.1	25.7	21.7	20.4
6-311++G(3df,3pd)	25.2	20.8	17.7	25.2	20.9	19.2	24.9	20.6	19.1	25.7	21.6	20.1
pc-2	25.7	21.3	18.4	25.3	21.2	19.8	25.0	20.9	19.3	26.2	22.2	21.2
pc-3	24.5	20.0	16.8	24.4	20.2	18.6	24.1	19.9	18.2	25.0	20.9	19.7
aug-pc-2	26.0	21.6	18.9	25.6	21.5	20.0	25.2	21.2	19.6	26.4	22.5	21.6
aug-pc-3	24.5	20.1	16.8	24.4	20.2	18.6	24.1	19.9	18.3	25.0	21.0	19.7
Def2-TZVP	24.1	20.0	17.7	23.9	20.3	19.2	23.6	20.0	18.9	25.0	21.5	20.8
Def2-TZVPD	25.1	20.9	18.4	24.6	21.0	19.9	24.3	20.7	19.4	25.7	22.1	21.5
cc-pVDZ	33.9	28.8	27.7	32.9	28.7	28.2	32.7	28.5	28.5	34.0	29.6	29.3
cc-pVTZ	24.9	20.4	17.6	24.9	20.7	19.2	24.6	20.4	18.8	25.6	21.8	21.0
cc-pVQZ	24.4	20.0	17.0	24.5	20.4	18.7	24.2	20.1	18.4	25.1	21.2	20.1
aug-cc-pVDZ	37.8	32.7	31.2	36.8	31.8	31.6	36.6	31.6	31.4	38.0	32.7	33.1
aug-cc-pVTZ	26.3	21.7	18.2	25.8	21.6	19.8	25.5	21.3	19.5	26.6	22.6	21.2
aug-cc-pVQZ	24.9	20.4	17.4	24.9	20.7	18.8	24.6	20.4	18.6	25.5	21.5	20.3

The Modified F1 Set Vibrational Modes

The F1 vibrational frequency benchmarking set of 1066 vibrations excludes atoms beyond the second periodic row, has no more than 4 non-hydrogen atoms per molecule, with no more than 10 atoms in total per molecule, and was derived from work by Shimanouchi[1-2] and Johnson et al,[3] including the most common isotopomer of each molecule. Hanson-Heine modified this set to include 894 vibrations by removing modes with significant multireference character,[4-5] internal rotational character, inversion character, and triple bond bending character.[6] Several of the experimental assignments have also been changed to match the more recent assignments used in the VIBFREQ1295 frequency set,[7] and the frequencies used here are reported in the table below to two decimal places. The vibrational frequencies for each molecule are listed below in units of wavenumber. The vibrational modes are numbered in order of ascending energy.

Table S7. Molecules and frequencies in Hanson-Heine's modified F1 vibrational set.

Molecule	Mode	ν (cm^{-1})
$AlCl_3$	1	151.00
	2	151.00
	4	375.00
	5	616.00
	6	616.00
B_2H_6	2	794.00
	3	833.00
	4	850.00
	5	915.00
	6	950.00
	7	973.00
	8	1012.00
	9	1177.00
	10	1180.00
	12	1768.00
	13	1915.00
	14	2104.00
	15	2524.00
	16	2525.00
	17	2591.00
	18	2612.00

BF_3	1	480.00
	2	480.00
	3	691.00
	4	888.00
	5	1449.00
	6	1449.00
BH_3CO	1	313.70
	2	313.70
	4	818.19
	5	818.19
	6	1083.10
	7	1115.00
	8	1115.00
	9	2166.00
	10	2387.00
	11	2456.00
	12	2456.00
C_2Cl_2	5	477.00
	6	993.02
	7	2234.00
C_2N_2	5	845.59
	6	2157.82
	7	2330.48
CH	1	2732.98
CH_2CCH_2	3	847.07
	4	847.07
	5	865.00
	6	998.80
	7	998.80
	8	1073.00
	9	1394.18
	10	1443.00
	11	1959.38
	12	3006.72
	13	3015.00
	14	3085.53
	15	3085.53
CH_2CCHCl	1	184.00
	3	548.00
	4	592.00
	5	767.00
	6	822.00
	7	875.00
	8	999.00

	9	1101.00
	10	1256.00
	11	1435.00
	12	1963.00
	13	3009.00
	14	3079.00
	15	3079.00
CH_2CCl_2	1	299.00
	2	372.00
	3	460.00
	4	603.00
	5	686.00
	6	800.00
	7	875.00
	8	1095.00
	9	1400.00
	10	1627.00
	11	3035.00
	12	3130.00
CH_2CH_2	1	826.00
	2	940.00
	3	949.00
	4	1027.00
	5	1220.00
	6	1344.00
	7	1444.00
	8	1630.00
	9	3012.00
	10	3014.00
	11	3084.00
	12	3105.00
$CH_2CHCHCH_2$	2	297.09
	3	512.20
	5	748.00
	6	888.80
	7	908.07
	8	909.50
	9	972.00
	10	990.30
	11	1013.37
	12	1204.20
	13	1277.80
	14	1296.20
	15	1380.60

	16	1442.20
	17	1596.45
	18	1644.30
	19	2984.00
	20	3010.00
	21	3011.40
	22	3012.50
	23	3099.00
	24	3100.63
CH_2CHCHO	2	323.82
	3	564.34
	4	593.08
	5	911.34
	6	958.74
	7	992.66
	8	1002.01
	9	1153.40
	10	1275.00
	11	1360.00
	12	1420.00
	13	1625.00
	14	1724.00
	15	2800.00
	16	2998.00
	17	3069.00
	18	3103.00
CH_2Cl_2	1	281.50
	2	712.90
	3	759.82
	4	898.66
	5	1153.00
	6	1268.86
	7	1435.00
	8	2997.66
	9	3055.00
$^3CH_2$	2	2992.00
	3	3213.00
CH_3CCCH_3	2	213.00
	3	213.00
	6	725.00
	7	1029.00
	8	1029.00
	9	1054.00
	10	1054.00

	11	1152.00
	12	1380.00
	13	1382.00
	14	1448.00
	15	1448.00
	16	1456.00
	17	1456.00
	18	2240.00
	19	2916.00
	20	2938.00
	21	2966.00
	22	2966.00
	23	2973.00
	24	2973.00
CH_3CCH	5	930.28
	6	1036.15
	7	1036.15
	8	1385.59
	9	1450.28
	10	1450.28
	11	2137.87
	12	2918.00
	13	2980.85
	14	2980.85
	15	3335.07
CH_3CH_2CN	5	786.00
	6	836.00
	7	1005.00
	9	1077.00
	10	1256.00
	11	1319.00
	12	1387.00
	13	1433.00
	14	1465.00
	15	1465.00
	16	2254.00
	17	2849.00
	18	2900.00
	19	2995.00
	20	3001.00
	21	3001.00
CH_3CH_2Cl	2	336.00
	3	677.00
	4	786.00

	5	974.00
	7	1081.00
	8	1251.00
	9	1289.00
	10	1385.00
	11	1448.00
	12	1448.00
	13	1463.00
	14	2881.00
	15	2946.00
	16	2967.00
	17	2986.00
	18	3014.00
CH_3CH_2F	2	415.00
	3	810.00
	4	881.00
	5	1050.00
	8	1274.00
	9	1365.00
	10	1397.00
	11	1449.00
	12	1466.00
	13	1491.00
	14	2920.00
	15	2947.00
	16	2965.00
	17	2985.00
	18	3000.00
CH_3CH_3	2	821.29
	3	821.29
	4	993.03
	5	1196.98
	6	1196.98
	7	1378.53
	8	1396.53
	9	1467.79
	10	1467.79
	11	1467.99
	12	1467.99
	13	2895.64
	14	2954.00
	15	2968.69
	16	2968.69
	17	2985.04

	18	2985.04
CH_3CHO	2	509.00
	3	767.15
	4	866.96
	6	1114.04
	7	1352.60
	8	1395.20
	9	1429.91
	10	1436.64
	11	1747.00
	12	2715.77
	13	2923.00
	14	2964.44
	15	3014.00
CH_3CN	3	919.92
	4	1040.79
	5	1040.79
	6	1390.00
	7	1448.03
	8	1448.03
	9	2266.45
	10	2953.92
	11	3040.69
	12	3040.69
CH_3COCH_3	3	385.00
	4	484.00
	5	530.00
	6	777.00
	7	877.00
	8	891.00
	9	1066.00
	10	1091.00
	11	1216.00
	12	1364.00
	13	1364.00
	14	1410.00
	15	1426.00
	16	1435.00
	17	1454.00
	18	1731.00
	19	2937.00
	20	2937.00
	21	2963.00
	22	2972.00

	23	3019.00
	24	3019.00
CH_3COF	2	420.00
	3	567.00
	4	598.00
	5	826.00
	6	1000.00
	7	1054.00
	8	1188.00
	9	1378.00
	10	1437.00
	11	1440.00
	12	1870.00
	13	2955.00
	14	3004.00
	15	3043.00
CH_3COOH	3	581.00
	4	642.00
	5	657.00
	6	847.00
	7	989.00
	8	1048.00
	9	1182.00
	11	1382.00
	12	1430.00
	13	1430.00
	14	1788.00
	15	2944.00
	16	2996.00
	17	3051.00
	18	3585.20
CH_3Cl	1	732.84
	2	1018.06
	3	1018.06
	4	1354.88
	5	1452.17
	6	1452.17
	7	2967.77
	8	3037.14
	9	3039.26
CH_3F	1	1048.61
	2	1182.67
	3	1182.67
	4	1459.39

	5	1467.81
	6	1467.81
	7	2916.64
	8	2998.44
	9	2998.44
CH_3NC	3	944.90
	4	1129.00
	5	1129.00
	6	1426.49
	7	1464.01
	8	1464.01
	9	2161.00
	10	2967.00
	11	3013.00
	12	3013.00
CH_3NH_2	3	1044.81
	4	1130.00
	5	1195.00
	7	1455.00
	8	1473.00
	9	1485.00
	10	1622.02
	11	2820.00
	12	2961.00
	13	2985.00
	14	3361.00
	15	3427.00
CH_3NNCH_3	3	312.00
	4	352.00
	5	589.00
	6	916.00
	7	1009.00
	8	1010.00
	9	1109.00
	10	1176.00
	12	1380.00
	13	1393.00
	14	1434.00
	15	1438.00
	16	1445.00
	17	1447.00
	18	1580.00
	19	2916.00
	20	2926.00

	21	2966.00
	22	2977.00
	23	2982.00
	24	2982.00
CH_3OCH_3	3	412.35
	4	933.91
	5	1103.95
	6	1150.00
	7	1179.00
	8	1227.00
	9	1244.00
	10	1452.00
	11	1452.00
	12	1464.00
	13	1464.00
	14	1464.00
	15	1464.00
	16	2817.00
	17	2817.39
	18	2925.00
	19	2952.00
	20	2996.00
	21	2996.00
CH_3OH	2	1033.70
	3	1070.20
	4	1156.50
	5	1335.20
	6	1453.30
	7	1474.20
	8	1483.50
	9	2844.70
	10	2961.10
	11	3003.70
	12	3682.50
CH_3SiH_3	2	524.02
	3	524.02
	4	702.98
	5	871.00
	6	871.00
	7	946.00
	8	946.00
	9	946.00
	10	1264.00
	11	1403.00

	12	1403.00
	13	2165.69
	14	2169.00
	15	2169.00
	16	2928.84
	17	2981.57
	18	2981.57
CH_4	1	1310.76
	2	1310.76
	3	1310.76
	4	1533.33
	5	1533.33
	6	2932.37
	7	3018.53
	8	3018.53
	9	3018.53
$CHCl_3$	1	260.59
	2	260.59
	3	367.30
	4	676.64
	5	775.96
	6	775.96
	7	1220.34
	8	1220.34
	9	3032.93
CHF_3	1	507.82
	2	507.82
	3	700.10
	4	1141.46
	5	1157.33
	6	1157.33
	7	1377.85
	8	1377.85
	9	3018.00
CO_2	1	667.38
	2	667.38
	3	1335.13
	4	2349.14
$COCl_2$	1	301.55
	2	443.17
	3	572.53
	4	582.09
	5	851.01
	6	1828.20

COClF	1	408.81
	2	501.59
	3	666.59
	4	764.39
	5	1095.06
	6	1875.83
COF_2	1	581.74
	2	619.08
	3	774.00
	4	963.32
	5	1243.27
	6	1944.61
COS	1	521.00
	2	521.00
	3	858.97
	4	2062.20
CS_2	1	395.99
	2	395.99
	3	672.73
	4	1535.36
CSF_2	1	417.00
	2	526.00
	3	622.00
	4	787.00
	5	1189.00
	6	1368.00
ClCN	3	747.82
	4	2249.25
F_2NH	1	500.00
	2	888.00
	3	972.00
	4	1307.00
	5	1424.00
	6	3193.00
F_2SO	1	376.00
	2	393.00
	3	530.00
	4	747.00
	5	808.42
	6	1334.00
FCN	3	1076.49
	4	2318.81
H_2CO	1	1167.26
	2	1249.09

	3	1500.17
	4	1746.01
	5	2782.46
	6	2843.33
H_2O	1	1594.74
	2	3656.65
	3	3755.79
H_2O_2	3	1266.00
	4	1402.00
	5	3599.00
	6	3608.00
H_2S	1	1182.58
	2	2614.41
	3	2628.45
HCCCCH	9	871.96
	10	2022.24
	11	2188.93
	12	3332.15
	13	3333.66
$HCCCH_2Cl$	7	908.00
	8	960.00
	9	1179.00
	10	1271.00
	11	1441.00
	12	2147.00
	13	2968.00
	14	3002.00
	15	3335.00
$HCCCH_2F$	6	940.00
	7	1018.00
	8	1039.00
	9	1242.00
	10	1381.00
	11	1465.00
	12	2150.00
	13	2955.00
	14	2972.00
	15	3338.00
HCCCl	5	750.36
	6	2113.85
	7	3339.36
HCCF	5	1061.45
	6	2239.20
	7	3356.96

HCCH	5	1974.34
	6	3288.58
	7	3372.82
HCN	3	2096.85
	4	3311.48
HCO	1	1080.76
	2	1868.17
$HCOOCH_3$	2	318.00
	4	767.00
	5	925.00
	6	1032.00
	7	1166.00
	8	1168.00
	9	1207.00
	10	1371.00
	11	1443.00
	12	1445.00
	13	1454.00
	14	1754.00
	15	2943.00
	16	2969.00
	17	3012.00
	18	3045.00
HCOOH	1	626.17
	2	640.73
	3	1033.47
	4	1109.20
	6	1379.05
	7	1776.83
	8	2942.00
	9	3568.90
HF	1	3959.00
HN_3	1	534.00
	2	607.00
	3	1151.00
	4	1264.00
	5	2140.00
	6	3336.00
HNCO	1	577.35
	2	660.00
	4	1322.25
	5	2268.89
	6	3538.25
HNO_3	2	580.30

	3	648.83
	4	763.15
	5	886.23
	6	1303.07
	7	1326.19
	8	1709.57
	9	3550.70
HOCl	1	724.36
	2	1238.62
	3	3609.48
HOF	2	1353.40
	3	3577.93
LiF	1	894.00
N_2	1	2330.00
N_2F_2	1	365.50
	2	423.00
	3	600.00
	4	990.00
	5	1010.00
	6	1522.00
N_2O	1	588.77
	2	588.77
	3	1284.90
	4	2223.76
NCl_2F	1	274.00
	2	344.00
	4	615.00
	5	692.00
	6	825.00
$NClF_2$	1	378.00
	2	382.00
	3	556.00
	4	697.00
	5	854.00
	6	930.00
NF_3	1	493.42
	2	493.42
	4	907.54
	5	907.54
NH	1	3125.57
NH_3	2	1626.28
	3	1626.28
	4	3323.71
	5	3443.68

	6	3443.68
NO_2	1	749.65
	2	1319.77
	3	1616.85
NSF	1	366.00
	2	642.00
	3	1372.00
O_2	1	1556.00
OCHCHO	2	341.52
	3	550.39
	4	801.48
	5	1047.91
	6	1065.00
	7	1312.51
	8	1338.00
	9	1732.10
	10	1745.00
	11	2835.07
	12	2844.00
OH	1	3568.00
ONF	1	519.60
	2	765.36
	3	1844.10
PCl_3	2	198.00
	4	482.00
	5	482.00
PF_3	1	347.09
	2	347.09
	4	859.22
	5	859.22
PH	1	2276.21
PH_3	2	1118.31
	3	1118.31
	4	2321.12
	5	2326.50
	6	2326.50
S_2F_2	1	182.50
	2	301.00
	3	319.80
	4	614.60
	5	680.80
	6	717.00
SH	1	2599.05
SO_2	1	517.87

	2	1155.71
	3	1362.06
SO_3	1	497.57
	2	530.09
	3	530.09
	4	1064.92
	5	1391.52
	6	1391.52
$SOCl_2$	2	283.73
	3	345.97
	4	459.82
	5	500.09
	6	1251.00
SiH	1	1971.00
SiH_2Cl_2	1	188.00
	2	527.00
	3	590.00
	4	602.00
	5	710.00
	6	876.00
	7	954.00
	8	2224.00
	9	2237.00
SiH_3CCH	3	659.00
	4	668.00
	5	668.00
	8	935.00
	9	946.00
	10	946.00
	11	2055.00
	12	2192.00
	13	2193.00
	14	2193.00
	15	3311.00
SiH_3Cl	1	550.93
	2	664.13
	3	664.13
	4	949.00
	5	949.00
	6	954.00
	7	2195.00
	8	2201.00
	9	2201.00
SiH_3F	1	729.00

	2	729.00
	3	875.00
	4	961.00
	5	961.00
	6	991.00
	7	2206.80
	8	2209.06
	9	2209.06
SiH_4	1	913.47
	2	913.47
	3	913.47
	4	974.60
	5	974.60
	6	2186.87
	7	2189.19
	8	2189.19
	9	2189.19
$SiHCl_3$	1	175.50
	2	175.50
	3	253.70
	4	498.60
	5	600.10
	6	600.10
	7	810.80
	8	810.80
	9	2260.90
$SiHF_3$	1	305.46
	2	305.46
	3	424.03
	4	843.60
	5	858.25
	6	858.25
	7	999.58
	8	999.58
	9	2315.60
cyclo-C_2H_4NH	1	773.00
	2	817.00
	3	856.00
	4	904.00
	5	998.00
	6	1090.00
	7	1095.00
	8	1131.00
	9	1211.00

	10	1237.00
	11	1268.00
	12	1463.00
	13	1482.00
	14	3015.00
	15	3015.00
	16	3079.00
	17	3079.00
	18	3338.00
cyclo-C_2H_4O	1	822.00
	2	860.00
	3	877.00
	5	1120.00
	6	1142.00
	7	1151.00
	9	1300.00
	10	1472.00
	11	1498.00
	12	3006.00
	13	3006.00
	14	3063.00
	15	3065.00
cyclo-C_3H_6	1	737.55
	2	737.55
	3	853.97
	4	868.36
	5	868.36
	6	1028.37
	7	1028.37
	8	1066.81
	9	1126.69
	10	1189.22
	11	1189.22
	12	1191.30
	13	1440.02
	14	1440.02
	15	1499.28
	16	3019.24
	17	3019.24
	18	3026.71
	19	3082.00
	20	3082.00
	21	3101.75
cis-CHClCHCl	1	173.00

	2	406.00
	3	571.00
	4	697.00
	5	711.00
	6	857.00
	7	876.00
	8	1179.00
	9	1303.00
	10	1587.00
	11	3072.00
	12	3077.00
cis-CHFCHF	1	255.00
	3	756.00
	4	768.00
	5	866.00
	6	1014.00
	7	1127.00
	8	1266.00
	9	1376.00
	10	1715.00
	11	3135.00
	12	3135.00
gauche-CH_2ClCH_2Cl	1	125.00
	2	272.00
	3	410.00
	4	669.00
	5	693.00
	6	890.00
	7	948.00
	8	1027.00
	9	1146.00
	10	1207.00
	11	1292.00
	12	1315.00
	13	1433.00
	14	1436.00
	15	2957.00
	16	2957.00
	17	3005.00
	18	3005.00
trans-CH2ClCH2Cl	2	228.00
	3	303.00
	4	712.00
	5	758.00

	6	769.00
	7	992.00
	8	1057.00
	9	1129.00
	10	1233.00
	11	1265.00
	12	1308.00
	13	1442.00
	14	1455.00
	15	2968.00
	16	2973.00
	17	3005.00
	18	3018.00
trans-CHClCHCl	1	227.00
	2	250.00
	3	350.00
	4	763.00
	5	828.00
	6	846.00
	7	900.00
	8	1200.00
	9	1274.00
	10	1578.00
	11	3073.00
	12	3090.00
trans-CHFCHF	1	329.00
	2	341.00
	3	548.00
	4	788.00
	5	875.00
	6	1123.00
	7	1159.00
	8	1274.00
	9	1286.00
	10	1694.00
	11	3111.00
	12	3114.00

Integration Grids.

The default Q-Chem integration grids have been used for each method. However, larger grids were necessary to converge some of the CH_3COOH and $HCOOCH_3$ molecules, particularly with meta-GGAs, and in these cases the Euler-Maclaurin-Lebedev EML-(99,590) integration grid was used. These grids are listed in the tables below.

Table S8. Default Q-Chem integration grids for each method.

Method	Integration Grid
HF	None
MP2	None
CCSD	None
BLYP	SG-1[8]
BP86	SG-1
EDF1	SG-1
HCTH-93	SG-1
HCTH-120	SG-1
HCTH-147	SG-1
HCTH-407	SG-1
KT3	SG-1
OLYP	SG-1
PBE	SG-1
B97M-V	SG-2[9]
rSCAN	SG-2
SCAN	SG-2
τ-HCTH	SG-2
TPSS	SG-2
VSXC	SG-2
B1LYP	SG-1
B3LYP	SG-1
B3P86	SG-1
B3PW91	SG-1
B97	SG-2
B97-K	SG-2
B97-1	SG-2
B97-2	SG-2
B97-3	SG-2
BHHLYP	SG-1
EDF2	SG-1
MPW1K	SG-1
MPW1PW91	SG-1
O3LYP	SG-1
PBE0	SG-1
X3LYP	SG-1
M05	SG-3[9]
M05-2X	SG-3
M06	SG-3
M06-2X	SG-3
M08-HX	SG-3
M08-SO	SG-3
τ-HCTHh	SG-2

TPSS0	SG-2
CAM-B3LYP	SG-1
HSE-HJS	SG-1
LC-rVV10	SG-1
ωB97X-D	SG-2
ωB97X-D3	SG-2
ωB97X-V	SG-2

Table S9. Calculations converged using the EML-(99,590) integration grid.

Method	Basis Set	Molecule
B97M-V	6-31++G(d,p)	CH_3COOH
B97M-V	6-31+G(2df,p)	CH_3COOH
B97M-V	6-31+G(d,p)	CH_3COOH
B97M-V	6-311++G(2df,p)	CH_3COOH
B97M-V	6-311++G(d,p)	CH_3COOH
B97M-V	6-311+G(2df,p)	CH_3COOH
B97M-V	6-311+G(d,p)	CH_3COOH
B97M-V	6-31G(d)	CH_3COOH
B97M-V	aug-cc-pVTZ	CH_3COOH
B97M-V	cc-pVTZ	CH_3COOH
τ-HCTH	6-311+G(d,p)	CH_3COOH
τ-HCTH	6-311++G(2df,p)	CH_3COOH
τ-HCTH	6-31+G(d,p)	CH_3COOH
τ-HCTH	6-31+G(2df,p)	CH_3COOH
τ-HCTH	aug-cc-pVTZ	CH_3COOH
τ-HCTH	6-311+G(2df,p)	CH_3COOH
τ-HCTH	6-311++G(d,p)	CH_3COOH
TPSS	6-311+G(d,p)	CH_3COOH
TPSS	6-311+G(2df,p)	CH_3COOH
TPSS	6-311++G(2df,p)	CH_3COOH
TPSS	6-311++G(d,p)	CH_3COOH
VSXC	6-311+G(2df,p)	CH_3COOH
VSXC	6-311++G(2df,p)	CH_3COOH
VSXC	cc-pVTZ	CH_3COOH
ωB97X-D	6-31++G(d,p)	$HCOOCH_3$
ωB97X-D	6-311++G(d,p)	$HCOOCH_3$
ωB97X-D	6-311++G(2df,p)	$HCOOCH_3$

References

1. Shimanouchi, T., Tables of Molecular Vibrational Frequencies Consolidated Volume I. National Bureau of Standards: Washington DC, 1972; p. 164.
2. Shimanouchi, T., Tables of Molecular Vibrational Frequencies Consolidated Volume II. *J. Phys. Chem. Ref. Data* **1977,** *6* (3), 993-1102.
3. Johnson, B. G.; Gill, P. M. W.; Pople, J. A., The performance of a family of density functional methods. *J. Chem. Phys.* **1993,** *98* (7), 5612-5626.
4. Hanson-Heine, M. W. D., Static correlation in vibrational frequencies studied using thermally-assisted-occupation density functional theory. *Chem. Phys. Lett.* **2020,** *739*, 137012.
5. Hanson-Heine, M. W. D., Static Electron Correlation in Anharmonic Molecular Vibrations: A Hybrid TAO-DFT Study. *J. Phys. Chem. A* **2022,** *126* (40), 7273-7282.
6. Hanson-Heine, M. W. D., Vibrational scaling factors follow a power law. *Spectrochim. Acta A* **2026,** *359*, 127967.
7. Zapata Trujillo, J. C.; McKemmish, L. K., VIBFREQ1295: A New Database for Vibrational Frequency Calculations. *J. Phys. Chem. A* **2022,** *126* (25), 4100-4122.
8. Gill, P. M. W.; Johnson, B. G.; Pople, J. A., A standard grid for density functional calculations. *Chem. Phys. Lett.* **1993,** *209* (5-6), 506-512.
9. Dasgupta, S.; Herbert, J. M., Standard grids for high-precision integration of modern density functionals: SG-2 and SG-3. *J. Comput. Chem.* **2017,** *38* (12), 869-882.
